# Discovery of a New Natural Product and a Deactivation of a Quorum Sensing System by Culturing a “Producer” Bacterium With a Heat-Killed “Inducer” Culture

**DOI:** 10.3389/fmicb.2018.03351

**Published:** 2019-01-17

**Authors:** Libang Liang, Amanda Sproule, Brad Haltli, Douglas H. Marchbank, Fabrice Berrué, David P. Overy, Kate McQuillan, Martin Lanteigne, Noelle Duncan, Hebelin Correa, Russell G. Kerr

**Affiliations:** ^1^Department of Chemistry, University of Prince Edward Island, Charlottetown, PE, Canada; ^2^Department of Biomedical Sciences, Atlantic Veterinary College, University of Prince Edward Island, Charlottetown, PE, Canada; ^3^Nautilus Biosciences Croda, Charlottetown, PE, Canada; ^4^Department of Pathology and Microbiology, Atlantic Veterinary College, University of Prince Edward Island, Charlottetown, PE, Canada

**Keywords:** natural product discovery, biotransformation, induction, quorum sensing, metabolomics

## Abstract

Herein we describe a modified bacterial culture methodology as a tool to discover new natural products via supplementing actinomycete fermentation media with autoclaved cultures of “inducer” microbes. Using seven actinomycetes and four inducer microbes, we detected 28 metabolites that were induced in UHPLC-HRESIMS-based analysis of bacterial fermentations. Metabolomic analysis indicated that each inducer elicited a unique response from the actinomycetes and that some chemical responses were specific to each inducer-producer combination. Among these 28 metabolites, hydrazidomycin D, a new hydrazide-containing natural product was isolated from the pair *Streptomyces* sp. RKBH-B178 and *Mycobacterium smegmatis*. This result validated the effectiveness of the strategy in discovering new natural products. From the same set of induced metabolites, an in-depth investigation of a fermentation of *Streptomyces* sp. RKBH-B178 and autoclaved *Pseudomonas aeruginosa* led to the discovery of a glucuronidated analog of the pseudomonas quinolone signal (PQS). We demonstrated that RKBH-B178 is able to biotransform the *P. aeruginosa* quorum sensing molecules, 2-heptyl-4-quinolone (HHQ), and PQS to form PQS-GlcA. Further, PQS-GlcA was shown to have poor binding affinity to PqsR, the innate receptor of HHQ and PQS.

## Introduction

Due to intensive research efforts focused on the discovery of microbial natural products over the past 60–70 years, it has become more challenging to identify novel structural classes of natural products (Watve et al., 2001). The decreased rate of discovery coupled with the increasing incidence of emerging and multi-drug resistant pathogens has made the need for discovering new natural products as pressing as ever (Davies, 2006). An important source of microbial natural products is filamentous bacteria belonging to the family Actinomycetales (Abdelmohsen et al., 2014, 2015). Among the actinomycetes, the genus *Streptomyces* is an especially rich source of natural products as 50% of all known antibiotics have been discovered from this taxonomic group (Kieser, 2000). Genomic analysis of streptomycetes and other filamentous members of the Actinomycetales have revealed that these organisms possess the genetic information to produce many more natural products than those are observed in laboratory cultures (Katz and Baltz, 2016). Consequently, there is great promise in unlocking the metabolic potential harbored in microbial genomes.

One approach that has been used extensively to enhance the diversity of metabolites produced by microorganisms is co-cultivation (Bertrand et al., 2014; Marmann et al., 2014). Under standard conditions actinomycetes are grown in an axenic state. While simplifying the fermentation and analysis process, this situation does not mimic the natural habitat of actinomycetes, which in nature are members of highly diverse microbial communities (Duncan et al., 2014). Co-cultivation aims to more closely mimic natural environments by exposing microorganisms to competition and chemical signaling that is absent under axenic conditions. Induction of natural product biosynthesis has been observed in bacteria-bacteria (Onaka et al., 2011; JiYoung and MyoungSug, 2012) and fungi-fungi (Degenkolb et al., 2002; Zhu et al., 2011; Huang et al., 2014; Wang et al., 2014) co-cultures. Interkingdom co-cultures of bacteria and fungi also commonly result in the induction of secondary metabolite biosynthesis in both the fungal (Cueto et al., 2001; Oh et al., 2005, 2007; Ola et al., 2013; Whitt et al., 2014) and bacterial (Yang, 2009; Zuck et al., 2011; Rateb et al., 2013) co-culture constituents.

The dynamics of co-cultures are challenging to characterize and in many cases the mechanisms by which silent gene clusters are activated are unknown. In some cases, direct cellular contact is required to induce production of otherwise silent metabolites (Schroeckh et al., 2009; Onaka et al., 2011). In other cases, direct contact is not required and the elicitor of silent metabolite biosynthesis is an apparently diffusible signal. For example, cell-free supernatants from cultures of *Pseudomonas aeruginosa* and three bacterial isolates from marine epiphytes enhanced antimicrobial activities of two marine bacteria isolated from the surface of a *Halichondria* sponge (Burgess et al., 1999). At least some of these diffusible signals are highly heat-stable as Mearns and co-workers showed that dialysates of live and heat-killed bacterial cultures were able to enhance antimicrobial activities of several marine bacteria (Mearns-Spragg et al., 1998). Similarly, Luti and co-workers showed that heat-killed bacterial and fungal cells induced phenazine production in *P. aeruginosa* (Luti and Yonis, 2013).

Standard co-culture strategies suffer from two significant drawbacks. Firstly, identifying the producer of induced metabolites can be challenging, especially when the metabolite is not detected under axenic culture conditions. Secondly, reproducibility can be difficult to achieve in co-cultures due to variable growth dynamics of the co-culture constituents. To simplify conventional co-culture methodology, we examined the effects of four different heat-killed inducer organisms (*Bacillus subtilis, P. aeruginosa, Mycobacterium smegmatis*, and *Aspergillus flavus*) on secondary metabolite profiles of seven actinomycetes using untargeted ultra-high performance liquid chromatography high-resolution electrospray ionization mass spectrometry (UHPLC-HRESIMS) metabolomic analysis. In this way, the co-culture system was simplified to a biochemical induction with a consistent inducer concentration and composition, instead of a biological induction with multiple fluctuating variables resulting from differences in growth rates and other biological and physical interactions between microbes. Using this methodology, we identified and unambiguously characterized a naturally occurring hydrazide and a new biotransformation of the *P. aeruginosa* quorum-sensing molecules, pseudomonas quinolone signal (PQS) and 4-hydroxy-2-heptylquinoline (HHQ), generated through a culture of a marine *Streptomyces* with autoclaved *M. smegmatis* and *P. aeruginosa*, respectively. To further evaluate the utility of the methodology, this approach was compared to the traditional OSMAC (One Strain Many Compound) approach, a culture-dependent method for natural product induction via altering culture conditions (Bode et al., 2002).

## Materials and Methods

### General Methods

All commercially available solvents and reagents were used without further purification. Deionized water was purified to 18.2 MΩ⋅cm on a Milli-Q^®^ Biocel water purification system (EMD Millipore). Automated reversed-phase medium pressure liquid chromatography (RP-MPLC) was performed on a Combi*Flash* Rf (Teledyne Isco) equipped with a photodiode array (PDA) detector. Reversed-phase high-performance liquid chromatography (RP-HPLC) was performed on an Accella^TM^ chromatography system (Thermo Fisher Scientific) equipped with a PDA and evaporative light scattering detector (ELSD Sedex 80). All ^1^H, ^13^C, and ^15^N NMR spectra were acquired on a 600 MHz Bruker Avance III NMR spectrometer operating at 600, 150, and 60 MHz, respectively. All chemical shifts were reported in δ units (ppm) and were referenced to the residual solvent signal (CD_3_OD: δ_H_ 7.26 ppm and δ_C_ 77.16 ppm; DMSO-*d_6_*: δ_H_ 2.50 ppm and δ_C_ 39.52 ppm, CDCl_3_: δ_H_ 7.26 ppm and δ_C_ 77.16 ppm; CD_3_CN: δ_H_ 1.94 ppm and δ_N_ 245.6 ppm). Coupling constants were reported in Hz with the following abbreviations: (s) singlet, (d) doublet, (t) triplet, (q) quartet, (quint) quintet, (m) multiplet. Tandem mass spectra were recorded on a Thermo LTQ Orbitrap Velos mass spectrometer using collision induced dissociation (CID). Optical rotations were recorded on an Autopol III polarimeter (Rudolph Research Analytical). Infrared spectra were acquired by attenuated total reflectance using a SMART iTR^TM^ accessory on a Nicolet^TM^ 6700 FTIR spectrometer (Thermo Fisher Scientific). Gas chromatography-mass spectrometry was operated on an Agilent 6890N Network GC system connected to a 5973 Inert Mass Selective Detector using 70 eV ionization voltage (EI).

Bacterial strains used in inducer-producer cultures and HHQ feeding experiments are listed in Table 1 and Supplementary Table S4, respectively. All actinomycetes and inducer strains (*B. subtilis* ATCC 6051, *A. flavus* NRRL 3357, *M. smegmatis* ATCC 12051 and *P. aeruginosa* ATCC 14210), were maintained on Difco ISP2 agar at 30°C. *P. putida* KT2440 [*pBBR-pqsR-pqsA’-‘lacZ*] (Müller and Fetzner, 2013) was maintained at 37°C on Luria-Bertani (LB) agar (Sambrook et al., 1989) supplemented with kanamycin (50 μg/mL; Sigma-Aldrich, 60615-5G).

**Table 1:** Microorganisms used in inducer-producer culture experiments.

Code	Strain (Accession No.)	Taxonomy (Blast Hit Accession No.)	Identity (%)	Source
**Producers**				
**I**	MF730-N6	*Kitasatospora griseola* MF730-N6	n.a.	1
**II**	NRRL B-16091	*Micromonospora aurantiaca*	n.a	2
**III**	RKAG-348 (KY362381)	*Streptomyces fulvissimus* (NR_103947.1)	99.86	3
**IV**	RKBH-B349 (KY362384)	*Streptomyces peucetius* (NR_024763.1)	99.39	3
**V**	RKND-616 (KY362395)	*Micromonospora maritima* (NR_109311.1)	99.26	3
**VI**	RKBH-B178 (KY362383)	*Streptomyces drozdowiczii* (NR_116093.1)	98.72	3
**VII**	M145	*Streptomyces coelicolor*	n.a	4
**Inducers**				
**PA**	ATCC 142105	*Pseudomonas aeruginosa*	n.a.	5
**BS**	ATCC 60515	*Bacillus subtilis*	n.a.	5
**MS**	ATCC 120515	*Mycobacterium smegmatis*	n.a.	5
**AF**	NRRL 33572	*Aspergillus flavus*	n.a.	2

*Sources of strains: (1) Japan International Patent Organism Depositary, (2) ARS culture collection (NRRL), (3) in house collection, (4) John Innes Institute, and (5) American Type Culture Collection. For actinomycetes obtained from our in-house culture collection, taxonomy was derived from the closest 16S rDNA match from a BlastN search of the GenBank 16S rRNA sequence database.*

For actinomycete fermentations, a two-stage seed culture process was used to generate inocula. Approximately a 1 cm^2^ region was scraped from the surface of a well grown agar plate and used to inoculate 7 mL of ISP2 broth in 25 mm × 150 mm culture tube containing 5 4-mm glass beads. The first-stage seed culture was incubated at 30°C with shaking (200 rpm) for 72 h after which 1 mL of the first-stage seed was then transferred to 7 mL of fresh ISP2 and cultured for an additional 24 h under the same conditions.

All cell lines were acquired from ATCC; MCF7 breast cancer cells and CCL-81 Vero kidney cells were grown and maintained in 15 mL of Eagle’s minimal essential medium (Sigma-Aldrich, M5650), HCT116 colon cancer cells in McCoy’s 5a Medium Modified (Sigma-Aldrich, M9309-500ML), and HTB26 breast cancer cells in Dulbecco’s Modified Eagle’s Medium/Nutrient Mixture F-12 Ham (Sigma, D6421-500ML) supplemented with 10% fetal bovine serum (VWR, CA95043-976). All cultures were supplemented with 100 μU penicillin and 0.1 mg/mL streptomycin (VWR, CA12001-692) and fermented in T-75 cm^2^ cell culture flasks at 37°C in a humidified atmosphere of 5% CO_2_. Cell culture medium was refreshed every 2–3 days and cells were not allowed to exceed 80% confluency.

### Bacterial Fermentation and Extraction

“Inducer” organisms were cultured on ISP2 agar (500 mL; static) or in ISP2 broth (500 mL; 200 rpm) for 2–6 days at 30°C. Incubation periods were 2 days for *B*. *subtilis* and *P. aeruginosa*, 4 days for *A. flavus*, and 6 days for *M. smegmatis*. Different incubation times were chosen due to the different growth rates of the four microorganisms. All cultures were heat killed by autoclaving at 121°C for 30 min. To prepare solid culture medium containing heat-killed inducer cultures, autoclaved cultures (agar or broth), without further filtration, were diluted with four equivalent volumes of freshly prepared ISP2 agar medium. The “no-inducer controls” were prepared by mixing autoclaved ISP2 broth or ISP2 agar with four equivalents of freshly prepared ISP2 agar. Media were distributed in Petri dishes (10 cm dia.).

Actinomycete inocula were prepare using a two-stage seed culture protocol (see above). Liquid cultures (200 μL) of actinomycete “producer” strains were spread on the surface of agar media and the plates were incubated at 30°C for 6 days. Duplicate experiments were performed. To extract metabolites from agar cultures, the agar was cut into ∼1 cm^2^ pieces and frozen at -80°C. After thawing the agar was extracted twice with 7 mL EtOAc. Extracts were combined, evaporated *in vacuo*, and analyzed by UHPLC-HRESIMS.

### UHPLC-HRESIMS Analysis

UHPLC-HRESIMS analysis was performed using an Exactive^TM^ Orbitrap mass spectrometer (Thermo Fisher Scientific) equipped with an Accela PDA (Thermo Fisher Scientific) and SEDEX Model 80 LT-ELSD detector (Sedere). The UHPLC was equipped with a Core Shell Kinetex C_18_ column (2.1 mm × 50 mm, 1.7 μm, Phenomenex), which was operated with a mobile phase flow rate of 0.5 mL/min and a linear gradient from H_2_O:CH_3_CN (95:5, 0.1% formic acid) at 0.2 min to 100% CH_3_CN (0.1% formic acid) at 4.8 min. The mobile phase was held at 100% CH_3_CN (0.1% formic acid) for 3.2 min before returning to H_2_O:CH_3_CN (95:5, 0.1% formic acid) over 0.5 min and equilibrating the column for 1.5 min. The mass spectrometer was operated in positive mode, monitoring a *m*/*z* range from 190 to 2000, using a resolution of 30,000, spray voltage of 2.5 kV, capillary temperature of 300°C, tube lens voltage of 145 V, skimmer voltage of 14 V, maximum injection time 50 ms, and microscans of 1. PDA detector was monitoring between 200 and 600 nm.

### Untargeted Metabolomics Analysis

Raw HRMS data was processed using MZmine 2 (Pluskal et al., 2010) as previously described, with minor modifications (Forner et al., 2013). Mass detection was performed using a noise level value (intensity) of 1 × 10^4^ counts/s (cps) and chromatogram building utilized a minimum time span of 0.1 min, a minimum peak height of 1E4 and a *m/z* tolerance of 0.001 *m/z* or 5 ppm. After chromatograms were built, chromatogram deconvolution was performed using the local minimum search algorithm to remove random signals. Chromatogram deconvolution parameters were as follows: chromatographic threshold – 35%, search minimum in RT range – 0.1 min, minimum relative height – 35%, minimum absolute height – 1E4, minimum ratio of peak top/edge – 1.5. Deconvoluted chromatograms were gap-filled using a *m/z* tolerance of 0.001 *m/z* or 5 ppm and deisotoped. Normalization was applied after deisotoping using the linear normalizer method in MZmine 2 using maximum peak intensity as the normalization type, and peak area as the peak measurement type. The normalized data were aligned and exported to CSV format as described previously (Forner et al., 2013).

The distribution of induced mass features between strains and treatments was visualized in a heat map created in R using the “d3heatmap” function. Replicate cultures were treated individually to assess reproducibility. Using the “colors” package, peak areas under 1E4 were colored white while areas between 1E4 and 1E8 were colored with a gradient of black to red, indicating mass intensity from low to high. Cluster analysis was performed with the “dendrogram” package by column (mass features) without further restriction.

### Feeding Experiments With Pure *P. aeruginosa* Metabolites

*Streptomyces* sp. RKBH-B178 inoculum was prepare using a two-stage seed culture protocol (see above). 210 μL of the second-stage seed was added in 7 mL of ISP2 and incubated at 30°C and 200 rpm. HHQ (Sigma-Aldrich, SML0747-10MG), PQS (Sigma-Aldrich, 94398-10MG), and rhamnolipids (R90 product, AGAE Technologies; 90% rhamnolipids A and B, 10% minor rhamnolipid congeners) were diluted in CH_3_OH and added to RKBH-B178 cultures (0–25 μM) after 48 h of growth. Cultures were incubated for four more days and then extracted twice with 7 mL EtOAc. Extracts were combined, dried under air and dissolved in 3 mL CH_3_OH supplemented with 20 μg/mL dioctyl phthalate (Sigma-Aldrich, D201154-5ML) as an internal standard for UHPLC-HRESIMS analysis. The experiment was performed in triplicate.

### Hydrazidomycin D Scale-Up, Purification and Structure Elucidation

*M. smegmatis* inducer was cultured on ISP2 agar for 6 days and then the culture was autoclaved. Fresh ISP2 agar and autoclaved *M. smegmatis* agar (mixed at 1:4 ratio) were used to prepare the culture medium upon which *Streptomyces* sp. RKBH-B178 was fermented in 300 agar plates (*d* = 15 cm, each plate contained 20 mL medium). After 6 days of incubation, the agar was cut into ∼1 cm^2^ pieces and stored at -80°C overnight and then extracted with about 6 L EtOAc overnight. Purification was first carried out using an automated flash chromatography system (Combiflash Rf, Teledyne ISCO) equipped with a PDA detector monitoring at 250 and 280 nm. The crude extract was prepared for solid load injection by adsorbing it onto C_18_ and the separation was performed with a 43 g C_18_ column (RediSep Rf Gold^®^) using a mobile phase flow rate of 40 mL/min. The mobile phase consisted of a linear gradient from CH_3_OH:H_2_O (1:9) to 100% CH_3_OH over 21 min followed by 100% CH_3_OH for 5 min. Hydrazidomycin D was further purified by RP-HPLC using a semi-preparative Gemini C6-Phenyl 110 Å column (250 mm × 10 mm, 5 μm; Phenomenex, 637824-1). An isocratic elution with 14% H_2_O and 86% CH_3_OH for 30 min was used. The eluent was monitored by ELSD and UV at 236 nm while the hydrazidomycin D was collected at 25.6 min.

The location of the double bond position in the C18 alkyl chain of hydrazidomycin D was determined using a modified dimethyl disulfide (DMDS) derivatization (Buser et al., 1983; Figure 2B). Hydrazidomycin D (250 μg) was dissolved in 2 mL methanolic HCl and refluxed with stirring overnight. The reaction was evaporated *in vacuo*. The methyl ester product sample was dissolved in 200 μL hexanes. 50 μL of neat DMDS (Sigma-Aldrich, 471569-25ML) was added followed by a 6% iodine solution in diethyl ether (5 μL) and incubated at 40°C overnight. The reaction was then diluted with 200 μL of hexanes before quenching with a 100 μL 5% Na_2_S_2_O_3_ solution in deionized water. The aqueous layer was extracted three times with 200 μL hexanes. The organic extracts were combined and dried under a stream of air. The sample was dissolved in 100 μL hexanes and analyzed by GC-EIMS. An Agilent 6890N Network GC system was operated using a HP-5MS column coated with 5% phenyl methyl siloxane (Agilent, 19091S-433). Helium was used as the carrier gas at a constant flow rate of 1.0 mL/min and the nominal initial pressure was 11.65 psi. The GC oven was programmed with an initial temperature of 120°C, which was held for three minutes. The temperature was increased at a rate of 17°C/min until it reached 280°C and then the oven was held at 280°C for 8 minutes. Agilent 5973 inert mass selective detector was used to monitor mass range from 50 to 400 amu with an electronic multiplier voltage of 1765 V.

*Hydrazidomycin D****(1)***: yellow oil; no UV absorbance; IR *ν*_max_ 3258, 3005, 2955, 2924, 1688, 1665, 1529, 1466, 1390, 1264 cm^-1^; for ^1^H and ^13^C NMR data see Supplementary Table S2; HRESIMS *m/z* [M+H]^+^ 421.3781 (calcd for C_26_H_49_N_2_O_2_, 421.3789).

### Production and Characterization of PQS-GlcA

*Streptomyces* sp. RKBH-B178 was fermented in 2.95 L (59 × 50 mL) of ISP2 broth containing 25 μM HHQ at 30°C and 200 rpm for 6 days. The fermentation broth was extracted with EtOAc and fractionated by an automated flash chromatography as described for hydrazidomycin D. PQS-GlcA was further purified by RP-HPLC using the same semi-preparative Gemini column as described previously. An isocratic elution with 55% H_2_O 0.1% formic acid (solvent A) and 45% CH_3_CN 0.1% formic acid (solvent B) over 15 min was used and PQS-GlcA was collected at 8.9 min. At 15 min, the column was cleaned with a linear gradient over 1 min to 100% solvent B, which was held for 10 min. The eluent was monitored by ELSD and UV at 238 and 321 nm.

The configuration of the GlcA moiety in PQS-GlcA was determined using Tanaka’s method (Tanaka et al., 2007; Wang et al., 2012). PQS-GlcA (100 μg) was dissolved in 10 μL tetrahydrofuran (THF) and 40 μL H_2_O. The acid hydrolysis was performed by adding 50 μL 2M HCl and incubating overnight at 95 °C. The reaction mixture was evaporated to dryness *in vacuo* and re-suspended in 50 μL pyridine containing 1 mg/mL D-cysteine methyl ester (D-CME, Sigma-Aldrich, C2174-1G). The mixture was sonicated until the crude product was dissolved and incubated at 60°C for 1 h. Then 0.5 μL neat *o*-tolyl isothiocyanate (Sigma-Aldrich, 253723-5G) was added and the reaction was stirred at 60°C for 1 h. The reaction mixture was evaporated to dryness *in vacuo* and the crude product was resuspended in CH_3_OH at a concentration of 0.5 mg/mL. Similarly, the corresponding derivatives from commercially available D-GlcA (Sigma-Aldrich, G5269-10G) were prepared using D- and L-CME (Sigma-Aldrich, 410209-5G) to obtain the appropriate standards for UHPLC-HRESIMS analysis; L-GlcA was not commercially available but the retention time of L-GlcA/D-CME was measured using its enantiomer D-GlcA/L-CME.

The crude products (10 μL) were analyzed by UHPLC-HRESIMS using a LTQ Orbitrap Velos mass spectrometer (Thermo Fisher Scientific) in positive mode. The UHPLC was equipped with a C_18_ Hypersil Gold 175 Å column (50 mm × 2.1 mm, 1.9 μm, Thermo Fisher Scientific, 25002-052130), which was operated with a mobile phase flow rate of 0.4 mL/min and a linear gradient from H_2_O:CH_3_OH (87:13, 0.1% formic acid) at 20.0 min to H_2_O:CH_3_OH (70:30, 0.1% formic acid) at 28.0 min, which was held for 2.0 min before starting a linear gradient to 100% CH_3_OH (0.1% formic acid) over 2.0 min. The mobile phase was held at 100% CH_3_OH (0.1% formic acid) for 6.0 min before returning to H_2_O:CH_3_OH (87:13, 0.1% formic acid) over 4.0 min and equilibrating the column for 2.8 min. Retention times of the reaction products were as follows: D-GlcA/D-CME 25.27 min, D-GlcA/L-CME 26.96 min, PQS-GlcA/D-CME 25.27 min.

*PQS-GlcA* (***6***): yellow solid; [*α*]^30^_D_ -252 (*c* 0.04, CH_3_OH); UV *λ*_max_ 228, 321 nm; IR *ν*_max_ 3278, 2929, 2814, 2188, 2036, 1611, 1517, 1352, 1115 cm^-1^; for ^1^H and ^13^C NMR data see Supplementary Table S3; HRESIMS *m/z* [M+H]^+^ 436.1966 (calcd for C_22_H_30_NO_8_, 436.1966).

### β-Galactosidase Assay

The quorum sensing activity of PQS-GlcA was determined using a *Pseudomonas putida* KT2440-based *LacZ* reporter strain [*pBBR-pqsR-pqsA’-‘lacZ*] kindly provided by Dr. Fetzner (Müller and Fetzner, 2013). *P. putida* was cultured in LB broth (1 mL) supplemented with 50 μg/mL kanamycin and the inoculation was adjusted to an OD_600_ of 0.05. HHQ, PQS, and PQS-GlcA in CH_3_OH were added to the cultures at concentrations between 0 and 20 μM. After incubating at 300 rpm for 5 h at 30°C, *β*-galactosidase activity was measured as described previously (Müller and Fetzner, 2013). *β*-galactosidase activity was expressed as Miller units using the equation_${}_{MU=\frac{1000\times \left(A_{420}-1.75\times A_{550}\right)}{OD_{600}\times V\times t}}$_. The experiment was performed in five replicates. Absorbance was measured using a SpectraMax M5e microplate reader (Molecular Devices). Dose-response curves were plotted using a built-in function log (agonist) vs. response (three parameters) and the EC_50_ values were calculated using $Y=Bottom+\frac{Top-bottom}{1+10^{LogEC_{50}-X}}$ to model non-linear regression in Prism 7.0 (GraphPad Software).

### Molecular Docking Simulations

Molecular docking simulations of PQS and PQS-GlcA with PqsR were performed using SwissDock (Grosdidier et al., 2011a,b). The crystal structure 4JVC (Ilangovan et al., 2013) was selected as the protein target. PQS and PQS-GlcA were submitted in MOL2 format as ligands. The computed binding results were viewed in Chimera (Pettersen et al., 2004) using default settings.

### Assessing HHQ Biotransformation in 14 Actinomycetes

Fourteen actinomycete isolates (Supplementary Table S4) from our culture collection were cultured in 7 mL of ISP2 broth containing 0, 10, and 100 μM HHQ for 6 days at 30°C, 200 rpm. Fermentations (in triplicate) were inoculated with 210 μL of a second stage seed culture and extracted twice with 7 mL of EtOAc. The EtOAc layers were combined and washed with H_2_O twice and the organic layer was evaporated to dryness *in vacuo*. Dried extracts were dissolved in 1 mL of CH_3_OH and analyzed by UHPLC-HRESIMS as described above.

### Antimicrobial Assay Against *M. smegmatis*

Hydrazidomycin D, rifampin (Sigma-Aldrich, R7382-1G), and isoniazid (Sigma-Aldrich, 13377-5G) were filter-sterilized separately and diluted to concentrations from 2.5 to 320 μg/mL in 20% DMSO. *M. smegmatis* was cultured on Difco Middlebrook 7H10 agar (Fisher Scientific, DF0627-17-4) for 4 days. The growth on the plate was swabbed to a seed tube containing 4.5 mL of autoclaved H_2_O and 10 4-mm glass beads, which was subsequently vortexed for 2 min and allowed to stand for 20 min. The suspended cells were used to adjust another seed tube of autoclaved H_2_O to the 0.5 McFarland Standard. 125 μL of the adjusted seed was added to 25 mL of Mueller-Hinton II (Cation-Adjusted) broth (Fisher Scientific, B11438) as inoculum. In a 96-well plate, each well was added 20 μL of the tested compounds, 100 μL of the inoculum, as well as another 80 μL of fresh Mueller-Hinton II (Cation-Adjusted) broth. Rifampin and isoniazid were used as antibiotic controls. Vehicle controls were prepared by 20% DMSO with Mueller-Hinton II (Cation-Adjusted) broth and media controls were only Mueller-Hinton II (Cation-Adjusted) broth. The experiment was performed in triplicate. After 6 days of stationary incubation at 30°C, growth or inhibition in each well was visualized by eye.

### Cytotoxicity Assays

The assay protocol was adopted from Overy et al. (2014). At 80% confluency, MCF7 breast cancer cells, HTB26 breast cancer cells, and HCT116 colon cancer cells were diluted and plated into 96-well cell culture plates at 5,000 cells/well, while CCL-81 Vero kidney cells were plated at 10,000 cells/well in 90 μL of respective culture media as described in General Methods. The plates were pre-incubated for 24 h before treatment with hydrazidomycin D (in 10 μL sterilize DMSO) at six final concentrations ranging from 32 to 1 μg/mL in triplicate. All cell lines were incubated at 37°C in a humidified atmosphere of 5% CO_2_ for 72 h. Each plate contained four uninoculated positive controls, four untreated negative controls, and one column containing a concentration range of zinc pyrithione or doxorubicin. Alamar blue was added 72 h after hydrazidomycin D addition to cell cultures (except 24 h for CCL-81 Vero kidney cells), and fluorescence was monitored using a Thermo Scientific Varioskan Flash plate reader at 560/12 excitation, 590 nm emission at time zero and 4 h after Alamar blue addition. The percentage of cell viability were calculated by subtracting the time zero emission measurement from the final reading and the IC_50_ value was determined by the lowest tested concentration reaching 50% cell inhibition.

### Metabolomic Comparison Between Addition of Heat-Killed Inducer and OSMAC Approaches

*Streptomyces* sp. RKBH-B178 was cultivated in 18 nutritionally diverse media composed of a range of carbon sources (simple and complex), nitrogen sources (inorganic, amino acid, and complex nitrogen sources), phosphate levels and osmolarity as well as ISP2 supplemented with *M. smegmatis* cell-free broth (CFB) or *M. smegmatis* cells. The recipes of these media for the OSMAC approach are listed in Supplementary Table S5.

To prepare the *M. smegmatis* culture, it was first cultured in ISP2 broth at 30°C and 200 rpm until confluent growth was observed. 25 mL of the seed culture was used to inoculate 2.8 L Fernbach flasks containing 500 mL of ISP2 broth. After 6 days at 30°C and 200 rpm, the *M. smegmatis* culture was autoclaved, and centrifuged at 10,000 x *g* for 10 min. The cells and CFB were separated and 1 mL of packed cells and CFB were diluted in 6 mL of fresh ISP2 to make the *M. smegmatis* CFB and cell media, respectively. All fermentations were conducted in 7 mL of medium in 25 mm × 150 mm tubes. Fermentations were inoculated with 3% (v/v) of a second stage seed culture and incubated at 30°C and 200 rpm for 6 days. The cultures were extracted by adding 7 mL EtOAc and shaking at 250 rpm for 1 h. The organic layer (5 mL) was removed and dried *in vacuo*. All samples were then resuspended in 3 mL CH_3_OH containing 10 μg/mL dioctyl phthalate as an internal standard and analyzed by UHPLC-HRESIMS. Xcalibur Software (Thermo Fisher Scientific) was used to integrate peak area of hydrazidomycin D across all the raw LCMS data files.

## Results

### Metabolomics Analysis of Cultures Treated With Heat-Killed Inducer

**FIGURE 1:**
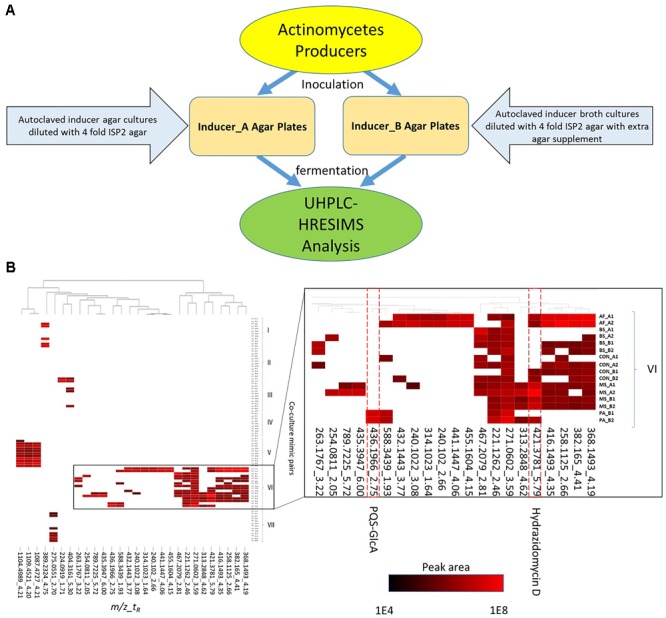
Inducer-producer culture strategy and metabolomic analysis. **(A)** Inducer-producer culture strategy overview. **(B)** UHPLC-HRESIMS based untargeted metabolomic analysis. 28 mass features are selected in the heatmap based on induction, upregulation, and peak area abundance. The vertical axis indicates actinomycete and inducer-producer pairings. Four inducer organisms include: PA, *P. aeruginosa*; BS, *B. subtilis*; MS, *M. smegmatis*; and AF, *A. flavus*. Preparation of the inducer organisms in ISP2 agar or broth is indicated by (A) or (B), respectively. The control experiments (CON) were prepared by adding twice-autoclaved ISP2 media to the mono-cultures of the producers. The two experimental replicates are indicated by 1 or 2. The horizontal axis indicates induced mass features (detected ions defined by a *m/z* and *t_R_* window) from UHPLC-HRESIMS analysis. The mass features are presented in a *m/z*_*t_R_* format and are clustered across different culture experiments. The white cells indicate peak areas <1E4, while peak areas from 1E4 to 1E8 were scaled from black (1E4) to red (1E8).

To evaluate the utility of the new culture strategy to modulate the secondary metabolism of actinomycetes, we utilized four phylogenetically distinct microbes as heat-killed “inducers” (*B. subtilis, A. flavus, M. smegmatis*, and *P. aeruginosa*) and evaluated their effect against seven actinomycete “producers” belonging to three different genera (*Streptomyces, Kitasatospora*, and *Micromonospora*) (Table 1). Inducers were chosen based on previous reports of interspecies induction of secondary metabolites in streptomycetes (Shank and Kolter, 2009; Onaka et al., 2011; Reen et al., 2011; Verheecke et al., 2015). Actinomycetes were cultured on agar supplemented with heat-killed inducer organisms (20% v/v) and extracts derived from these cultures were analyzed by UHPLC-HRESIMS to identify metabolites whose production was induced in response to exposure to heat-killed inducer organisms (Figure 1A).

In total, 890 mass features were identified after processing of the UHPLC-HRESIMS raw data with a mass intensity threshold of 1E4. After removing mass features detected in media blanks and monocultures of inducers, 659 mass features remained. These mass features represented metabolites produced by the actinomycetes in standard conditions and those produced in response to the presence of autoclaved inducers. The relative increase in production of these metabolites was examined by comparing mass features’ peak areas between the standard and treated cultures. In this analysis, 20 mass features showed a more than five-fold increase (up-regulated) in at least one inducer-producer pair. In parallel, another 17 induced mass features were highlighted because they were absent in the standard conditions (induced) and their peak areas were higher than 1E5 in at least one inducer-producer pair. To reduce redundancy in the 37 mass features, the [M+Na]^+^ and [M+NH_4_]^+^ adducts were deleted if their corresponding [M+H]^+^ adducts were detected. The final dataset contained 28 mass features of interest including up-regulated mass features and induced mass features (Supplementary Table S1).

To visualize patterns of metabolite induction, a heat map showing the distribution of the 28 mass features of interest across strains and treatments was constructed (Figure 1B). Cluster analysis of the induced metabolite profiles revealed clustering based primarily on actinomycete strain and type of inducer treatment, indicating that each actinomycete exhibited a unique response to the inducers (Figure 1B). Strain VI (*Streptomyces* sp. RKBH-B178) exhibited the highest response in the cultures with 21 mass features of interest. Strains III (*Streptomyces* sp. RKAG-348) and VI showed metabolic responses to agar-derived *A. flavus*, whereas the other producer strains had poor or no growth. Strain VI also had a unique response to broth-derived *P. aeruginosa*, while all other actinomycetes had poor or no growth. This inhibition was presumably due to the production of growth inhibitory substances by the inducer strains. Known toxins such as aflatoxins from *A. flavus* and pyocyanin from *P. aeruginosa* were detected by UHPLC-HRESIMS in broth and agar extracts at various levels (data not shown). Strain VI exhibited resistance to these inhibitors that otherwise restricted the growth of the other producer strains, although the mechanism of this resistance was not investigated.

To validate this approach and determine if this strategy was an effective method of altering, and ultimately enriching, actinomycete secondary metabolomes, we attempted to identify the induced metabolites shown in Figure 1B by comparison of observed *m*/*z* values (exact masses) to those contained in a commercial database AntiBase (Laatsch H. Antibase: The Natural Compound Identifier. Wiley-VCH, 2017 version). Subsequent isolation and spectroscopic analysis led to the identification of a new hydrazide and a new biotransformation product of quorum sensing molecules of *P. aeruginosa.*

### Identification of a New Hydrazide From the Cultivation of *Streptomyces* sp. RKBH-B178 With Heat-Killed *M. smegmatis*

Strain VI (*Streptomyces* sp. RKBH-B178) was observed to produce the highest number of mass features. In these fermentations we observed a unique mass feature (421.3781_5.79), which was up-regulated by agar-derived *A. flavus* and agar- and broth-derived *M. smegmatis*. The highest production was achieved when treated with agar-derived *M. smegmatis* (Figure 1B), resulting in an 18.7-fold increase in peak area over control. No natural product with a *m/z* value within 5 ppm was found in AntiBase, suggesting this metabolite was putatively new. To purify the metabolite, a 6 L scale-up fermentation was performed using the heat killed inducer addition with agar-derived *M. smegmatis*. The crude extract was then subjected to automated flash chromatography and HPLC purification to afford **1** hydrazidomycin D (0.5 mg), which was named based on structural similarities to hydrazidomycins (Ueberschaar et al., 2011).

**FIGURE 2:**
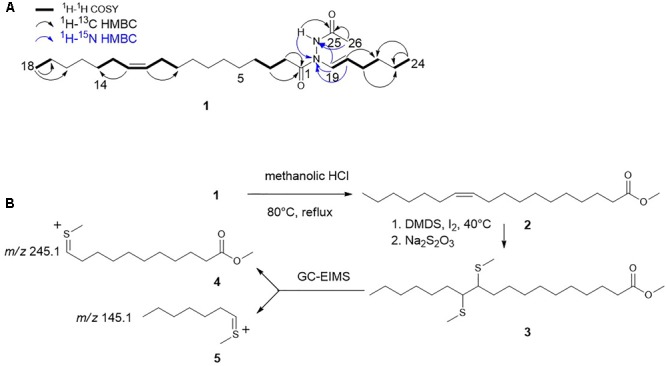
Structural characterization of hydrazidomycin D **(1)**. **(A)** Hydrazidomycin D key COSY, ^1^H-^13^C HMBC, and ^1^H-^15^N HMBC (H→C) correlations. **(B)** Overall scheme of DMDS derivatization to determine the position of the double bond in the C18 alkyl chain.

The molecular formula of **1** was established on the basis of HRESIMS analysis (*m/z* 421.3781 [M+H]^+^) to be C_26_H_48_N_2_O_2_ (Supplementary Figure S3A), indicating four degrees of unsaturation. The IR spectrum suggested the presence of two carbonyl groups with stretches at 1665 and 1688 cm^-1^. The ^1^H NMR spectrum indicated the presence of a double bond comprised of olefinic resonances H-19 (δ_H_ 7.01) and H-20 (δ_H_ 4.94). The large vicinal coupling constant (*J* = 14.1 Hz) across the Δ^19,20^ double bond allowed the *E* configuration to be assigned. The structure of the linear six-carbon alkyl chain was then confirmed by COSY correlations between H-21 (δ_H_ 1.99) and H-20 (δ_H_ 4.94) /H-22 (δ_H_ 1.28) and HMBC correlations between H-24 (δ_H_ 0.85) and C-22 (δ_C_ 31.6)/C-23 (δ_C_ 21.5) (Figure 2A and Supplementary Figure S1). The ^1^H-^15^N HMBC spectrum showed correlations between H-19 and both N^α^ (δ_N_ 134.6) and N^β^ (δ_N_ 151.2), suggesting the presence of a hydrazide moiety. A ^1^H-^15^N HMBC correlation between H-20 and N^β^ confirmed a covalent bond between C-19 (δ_C_ 123.9) and N^β^ (Figure 2A and Supplementary Figures S2A,C). An acetate moiety was also deduced from methyl proton H-26 (δ_H_ 1.97) and a HMBC correlation between H-26 and C-25 (δ_C_ 168.1). The incorporation of this acetate moiety at N^α^ was established from the ^1^H-^15^N HMBC correlation between H-26 and N^α^. The ^1^H-^15^N HSQC spectrum confirmed the attachment of hydrazide proton H-N^α^ (δ_H_ 8.41) to N^α^ (Figure 2A and Supplementary Figures S2A,B). An additional ^1^H-^13^C HMBC correlation between H-N^α^ and C-25 and a ^1^H-^15^N HMBC correlation between H-N^α^ and N^β^ confirmed the assignment of the acetylhydrazide moiety (Figure 2A and Supplementary Figures S2A,C).

Meanwhile, an unsaturated fatty acid moiety was established based on the presence of methylene protons H-2 (δ_H_ 2.31/2.09) /H-3 (δ_H_ 1.45), methyl proton H-18 (δ_H_ 1.26), and olefinic protons H-11/H-12 (δ_H_ 5.32). The apparent lack of methine protons and carbon signals suggested a linear fatty acid chain with no branching, while the proposed molecular formula indicated a chain length of 18 carbons. Methylene protons H-2 and H-3 exhibited HMBC correlations to carbonyl C-1 (δ_C_ 171.6). The connectivity between C-1 and N^β^ could not be unambiguously assigned on the basis of two-dimensional NMR, owing to the apparent lack of a ^1^H-^15^N correlation between H-2 and N^β^ and ^1^H-^13^C correlations between H-N^α^/H-19 and C-1. However, the lack of other positions on the acetylhydrazide moiety for the attachment of the fatty acid moiety indicates the connectivity must be between C-1 and N^β^. Structurally similar natural products geralcin B (Le Goff et al., 2012), geralcin C, and geralcin E (Le Goff et al., 2013) were reported without these same correlations, indicating the correlation might not be able to observed using conventional NMR techniques. The configuration of the Δ^11,12^ double bond was assigned on the basis of the chemical shifts of allylic carbon resonances C-10/C-13 (δ_C_ 27.4 in CDCl_3_, Supplementary Figure S1F), indicating the *Z* configuration (van Boom et al., 1977). The proposed structure of hydrazidomycin D (Figure 2A) was further corroborated by tandem mass spectrometry with the observation of a fragment ion with *m/z* of 157.1331, consistent with fragmentation across the amide moiety of C-1 and N^β^ (Supplementary Figure S3B).

The position of the Δ^11,12^ double bond in the 18-carbon alkyl chain was unambiguously determined by GC-EIMS analysis of the DMDS derivative of its fatty acid methyl ester (**3**). The parent ion with a *m/z* of 390.2 [M^+^]^+^ underwent fragmentation to generate daughter ions with *m/z* values of 245.1 (**4**) and 145.1 (**5**). This observation is consistent with fragmentation across the C-11/C-12 bond, which is between the two thiomethyl groups (Supplementary Figure S3C), revealing the position of the double bond and indicating the presence of a *cis*-vaccenic acid moiety in hydrazidomycin D. Pure hydrazidomycin D showed an IC_50_ value of 16 μg/mL against MCF7 breast cancer cells and was not active against CCL-81 Vero kidney cells, HTB26 breast cancer cells, HCT116 colon cancer cells. Further, it was inactive against the inducer *M. smegmatis* up to 32 μg/mL.

### Identification of a New Metabolite From the Cultivation of *Streptomyces* sp. RKBH-B178 With Heat-Killed *P. aeruginosa*

**FIGURE 3:**
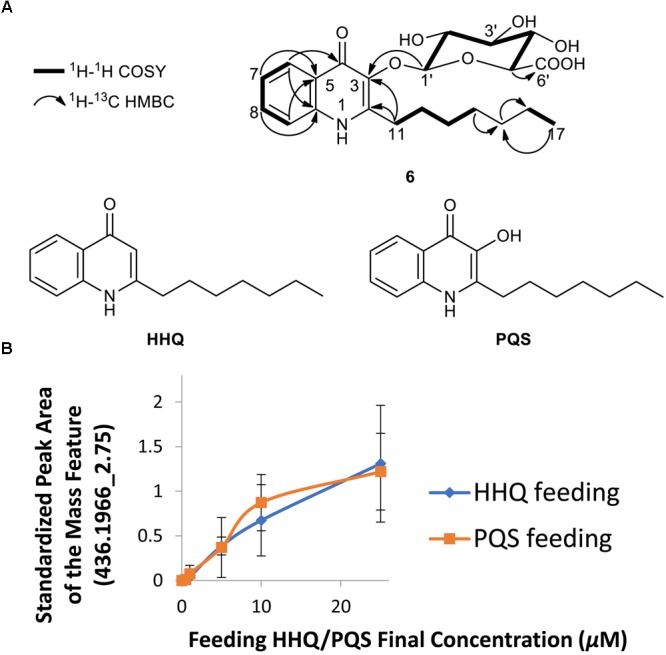
Biotransformation of two quorum sensing molecules of *P. aeruginosa*. **(A)** Chemical structures of quinolones PQS-GlcA **(6)**, HHQ, and PQS. Key COSY and HMBC (H→C) correlations for PQS-GlcA are shown. **(B)** Induction of the metabolite *m/z* 436.1966 [M+H]^+^, *t_R_* 2.75 min from *Streptomyces* sp. RKBH-B178 treated with different concentrations of HHQ and PQS, experiments conducted in triplicate. The peak area was calculated from integration of UHPLC-HRESIMS data. Peak areas were standardized using an internal standard (20 μg/mL dioctyl phthalate) added post-extraction to each sample.

We also chose to explore the *P. aeruginosa*-*Streptomyces* sp. RKBH-B178 inducer-producer pair, where we observed a unique mass feature (436.1966_2.75) with a peak area of 1E4 in the UHPLC-HRESIMS dataset in both replicates (Figure 1). No natural products with a *m/z* value within 5 ppm were found in AntiBase 2017, suggesting this metabolite (**6**) was putatively new. To investigate potential triggers for the biosynthesis of **6**, we analyzed extracts of autoclaved *P. aeruginosa* by UHPLC-HRESIMS and identified the major metabolites as HHQ, PQS, rhamnolipids and possible analogs of HHQ and rhamnolipids (Supplementary Figures S4A,B). Therefore, we tested the ability of commercially available HHQ, PQS and rhamnolipids to induce the production of metabolite **6** in *Streptomyces* sp. RKBH-B178. The target metabolite was only observed when RKBH-B178 was treated with various concentrations of HHQ and PQS (Figure 3B), suggesting HHQ and PQS from the *P. aeruginosa* cultures were responsible for the production of the target compound. Addition of rhamnolipids was not effective in inducing this metabolite.

Extraction of a scale-up fermentation (2.95 L) of RKBH-B178 treated with 25 μM HHQ afforded 409 mg of crude extract, which was fractionated by automated flash chromatography and subsequently purified by RP-HPLC, providing 0.4 mg of pure compound **6**. UHPLC-HRESIMS analysis of compound **6** supported a molecular formula of C_22_H_29_NO_8_ (*m/z* 436.1966 [M+H]^+^), indicating eight degrees of unsaturation. The NMR data (Supplementary Table S3 and Supplementary Figure S5) revealed the presence of four aromatic protons, H-6 (δ_H_ 8.26), H-7 (δ_H_ 7.37), H-8 (δ_H_ 7.67), and H-9 (δ_H_ 7.62), which were assigned by interpretation of COSY correlations and key HMBC correlations H-6/C-10 (δ_C_ 140.0), H-9/C-5 (δ_C_ 126.2), H-7/C-5 and H-8/C-10 (Figure 3A). The HMBC correlation between H-6 and C-4 (δ_C_ 174.0) allowed one of the two carbonyl resonances to be unambiguously assigned to the C-4 position. The presence of a seven-carbon alkyl chain was identified using COSY and HMBC correlations suggesting structural similarity to HHQ and PQS (Figure 3A). The HMBC correlations H-11 (δ_H_ 3.22, 2.99)/C-2 (δ_C_ 151.6), and H-11/C-3 (δ_C_ 139.1) allowed the two remaining aromatic carbon resonances to be assigned, indicating the presence of a quinolone ring with the alkyl chain attached to the C-2 position. The spectroscopic data is in agreement with that previously reported for both HHQ and PQS (Reen et al., 2012).

The characteristic anomeric carbon resonance C-1′ (δ_C_ 107.6) and the HMBC correlation between anomeric proton H-1′ [4.68 (d, *J* = 7.8 Hz)] and C-3 revealed the presence of a carbohydrate moiety attached to the C-3 position of the quinolone ring. The glycosidic spin system H-1′ through to H-5′ was assigned by interpretation of COSY correlations. The remaining carbonyl signal was assigned to the C-6′ position on the basis of an HMBC correlation H-5′ (δ_H_ 3.57)/C-6′ (δ_C_ 177.2) and is consistent with the proposed molecular formula. Further, coupling constants exhibited by H-1′ and H-4′ [δ_H_ 3.53 (t, *J* = 9.3 Hz)] showed their axial relationship with neighboring protons suggesting the presence of a *β*-glucuronic acid moiety. The configuration of the carbohydrate substituent was determined using Tanaka’s method (Tanaka et al., 2007; Wang et al., 2012), which confirmed that the sugar was D-glucuronic acid (Supplementary Figure S5F). Furthermore, tandem mass spectrometry (Supplementary Figure S6) revealed fragmentation between the glucuronic acid (GlcA) moiety and the aglycone, further corroborating the proposed structure of **6** as glucuronidated PQS (PQS-GlcA).

### Inactivation of HHQ/PQS by Glucuronidation

**FIGURE 4:**
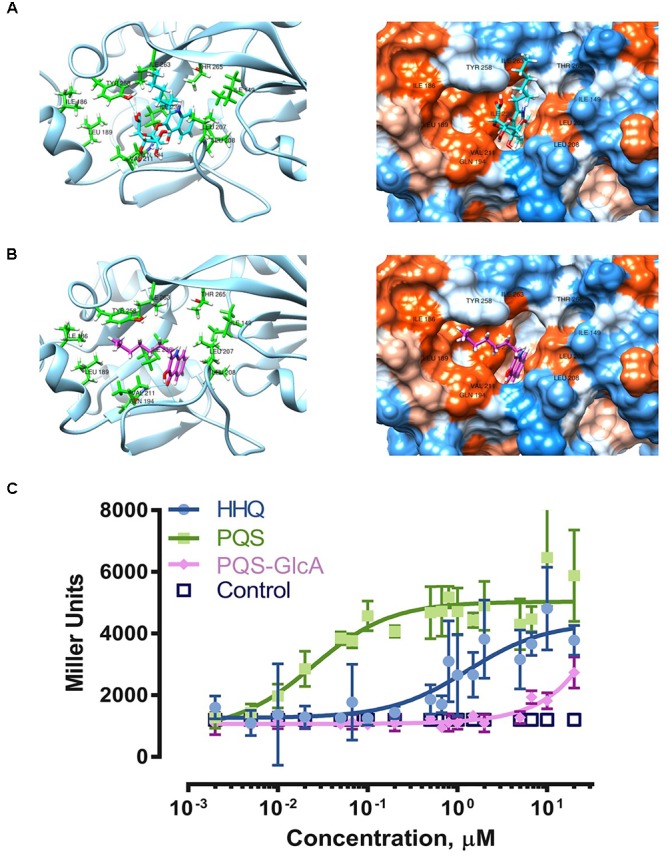
Biotransformation and its effects on PQS-PqsR quorum sensing. Molecular docking of PQS-GlcA **(A)** and PQS **(B)** with PqsR^CBD^. Images on the left are ribbon representations and images on the right are hydrophobicity surface representations. Visualization was in Chimera (Pettersen et al., 2004). **(C)** Experimental comparison of quorum sensing activities of HHQ, PQS, and PQS-GlcA using the reporter strain *Pseudomonas putida* KT2440 [pBBR-pqsR-pqsA’-‘lacZ] (Müller and Fetzner, 2013). The results are expressed as mean ±*SD* (*N* = 5).

Since HHQ/PQS are important quorum sensing molecules of the opportunistic pathogen *P. aeruginosa*, we further investigated the impact of the biotransformation on HHQ/PQS quorum sensing activities. Analysis of the PqsR^CBD^- NHQ (2-nonyl-4-hydroxyquinoline) complex crystal structure showed that the bicyclic ring of NHQ fits into the deep “B pocket” of PqsR^CBD^ defined by the residues Leu207, Leu208 and Ile236, while the alkyl side chain of NHQ fits into the “A pocket” with Tyr258 pinning the alkyl chain against Ile186, Val170, Leu189 and Ile236 (Ilangovan et al., 2013). Ilangovan et al. suggested that the binding of HHQ and PQS should orient in a similar way to NHQ due to structural similarities of these quinolone analogs. Modeling of PQS and PQS-GlcA in the PqsR^CBD^ active site (PDB - 4JVC) (Ilangovan et al., 2013) revealed computed clusters of PqsR^CBD^-PQS (cluster 2) and PqsR^CBD^-PQS-GlcA (cluster 29) in which the ligands adopted a similar orientation as NHQ in PqsR^CBD^ (Figures 4A,B). As shown in the ribbon diagram, PQS-GlcA is unable to orient its alkyl chain in the same fashion as PQS. From the hydrophobicity surface representation, the additional stearic bulk of glucuronic acid prevents the bicyclic ring from fully inserting into the “B pocket.” The “FullFitness” values from SwissDock, by averaging 30% of the total energy of the system and the solvation energy of PQS and PQS-GlcA were calculated to be -1161.66 and -1086.10 kcal/mol, respectively, suggesting the PQS-GlcA would have a lower affinity for PqsR. These modeling experiments suggest that PQS glucuronidation would hinder binding of PQS-GlcA to PqsR and may therefore adversely affect the PQS/HHQ quorum sensing system in *P. aeruginosa.*

To experimentally determine the effect of PQS glucuronidation on binding activity with its innate receptor PqsR, we compared the ability of PQS, HHQ, and PQS-GlcA to activate PqsR controlled gene expression using a previously developed reporter strain, *Pseudomonas putida* KT2440 [*pBBR-pqsR-pqsA’-‘lacZ*] (Müller and Fetzner, 2013). In this strain *pqsR* was constitutively expressed under the control of the *lac* promoter while the *pqsA* promoter and a portion of *pqsA* (-245 to +195 region) was fused in frame to a 5′-truncated *lacZ* reporter gene. Binding of PQS and HHQ to PqsR facilitates activation of the *pqsA* promoter and expression of *β*-galactosidase (LacZ).

Our results clearly show potent activation of PqsR controlled gene expression by PQS and HHQ (Figure 4C). The EC_50_ of PQS was 0.025 μM with 95% confidence interval (CI_95_) between 0.013 and 0.047 μM, which was 53-fold more potent than HHQ (EC_50_ 1.329 μM, CI_95_ 0.710 – 2.56 μM). These results were similar to those previously reported using this reporter system (Müller and Fetzner, 2013), although the EC_50_ of PQS was six-fold lower in our assay. Overall these results are consistent with previous assessments of PQS and HHQ quorum sensing activity, which have shown that HHQ is less active than PQS (Wade et al., 2005; Xiao et al., 2006). In comparison, PQS-GlcA exhibited minimal quorum sensing activity with an extrapolated EC_50_ of 1600 ± 8 μM. These results clearly demonstrate that glycosylation of PQS abolishes its PqsR binding activity.

To assess the generality of the transformation of HHQ to PQS-GlcA by actinomycetes, we cultured 14 additional taxonomically diverse actinomycete strains (Supplementary Table S4) in the presence of HHQ. Interestingly, although oxidation of HHQ was detected in different species, none of these additional 14 actinomycetes biotransformed HHQ to PQS-GlcA (data not shown).

### Metabolomic Comparison of Cultures Treated With Heat-Killed Inducer and OSMAC Approaches

*Streptomyces* sp. RKBH-B178 was chosen as the model producer and cultivated in 18 nutritionally diverse media composed of a range of carbon sources (simple and complex), nitrogen sources (inorganic, amino acid, and complex nitrogen sources), phosphate levels and osmolarity as well as ISP2 supplemented with *M. smegmatis* cell-free broth (CFB) or centrifuged *M. smegmatis* cells. Hydrazidomycin D was only produced in *M. smegmatis* cell-supplemented ISP2 medium, not in any other medium.

## Discussion

The new bacterial fermentation strategy coupled with metabolomic analysis employed in this study efficiently identified metabolites elicited through exposure of actinomycetes to autoclaved cultures of three bacteria and one fungus. A benefit of this approach is that induced metabolites can readily be ascribed to the “producer” actinomycetes, as metabolites originating from “inducers” are electronically filtered out of the dataset. The autoclaved culture as a whole was used in our experiments, whereas previous modified co-culture strategies used only inducer cells by employing an extra step of centrifugation (Luti and Mavituna, 2011; Luti and Yonis, 2013; Wang et al., 2013) or dialysis (Mearns-Spragg et al., 1998). While autoclaving may lead to degradation of some metabolites and cellular components, the autoclaved inducers were still able to induce metabolic changes in the actinomycetes tested. While we did not exhaustively characterize the effect of autoclaving on all inducer preparations, no major differences were observed between UHPLC-HRESIMS profiles of *P. aeruginosa* and *M. smegmatis* extracts pre- and post-autoclaving (Supplementary Figure S4). Using autoclaved inducers rather than filtered or dialyzed culture broths also preserves large cellular components (e.g., cell wall fragments), which may play an important role in triggering metabolic shifts (König et al., 2013; Schroeckh et al., 2009). Since large quantities of heat-killed inducer can be prepared and stored frozen for several months, inter-experiment variation can be reduced. Furthermore, the use of multiple inducer strains is an effective strategy to increase the diversity of metabolites produced by actinomycetes as we observed distinct patterns of metabolite production in response to different inducer strains. Overall, this fermentation strategy appears to be an effective and reproducible approach to modulating actinomycete secondary metabolism.

A comparison of inducer-producer fermentations with the traditional OSMAC approach using 18 different media was undertaken, and it was determined that only treatment with *M. smegmatis* resulted in production of **1**. The OSMAC approach in this case was ineffective in inducing this new metabolite. Further, *M. smegmatis* cells and CFB were added separately to *Streptomyces* sp. RKBH-B178 in ISP2. Only cultures treated with centrifuged *M. smegmatis* cells were found to produce hydrazidomycin D indicating the apparent requirement for an interaction with a cell surface component and providing evidence that our new strategy of bacterial culture can be an effective route to natural product discovery.

The example of the increased production of hydrazidomycin D (**1**) demonstrated how the combination of the new culture strategy and high-throughput UHPLC-HRESIMS-based metabolomics enabled expansion of microbial chemical space for natural product discovery. Although **1** was produced in trace amounts under standard cultivation conditions, treatment of *Streptomyces* sp. RKBH-B178 with autoclaved agar-derived *M. smegmatis* increased the yield of **1** by 18.7 fold. This level of up-regulation was essential for the isolation and characterization of **1**.

Natural products containing N-N single bonds are rare in nature, and therefore, their biosynthesis and biological functions remain unclear. A recent study suggested three enzymes responsible for hydrazine biosynthesis and the encoding genes were commonly found in bacteria (Matsuda et al., 2018). Compounds structurally similar to hydrazidomycin D with nitrogen-nitrogen bonds have been shown to exhibit antimicrobial and anticancer activities (Supplementary Table S5). For example, hydrazidomycin A and B had average IC_50_ values of 0.857 and 10.7 μM, respectively against 42 human tumor cells (Ueberschaar et al., 2011). Geralcin B has a reported IC_50_ value of 5 μM against MDA231 breast cancer cells (Le Goff et al., 2012), while geralcin C had an IC_50_ value of 0.8 μM against KB and HCT116 cancer cells (Le Goff et al., 2013). Elaiomycin B and C were weakly active against *S. lentus* with IC_50_ value of 100 μM for both compounds, but moderate active against acetylcholinesterase (IC_50_ value of 1.0 and 2.0 μM, respectively) and phosphodiesterase (IC_50_ value of 6.5 and 8.5 μM, respectively) (Helaly et al., 2011; Kim et al., 2011). Hydrazidomycin D, however, was weakly active against MCF7 breast cancer cells (IC_50_ value of 16 μg/mL), and was not active against CCL81 Vero kidney cells, HTB26 breast cancer cells, or HCT116 colon cancer cells up to 32 μg/mL. We also tested hydrazidomycin D for antimicrobial activity against its inducer *M. smegmatis*, but it was inactive, suggesting the increased production by hydrazidomycin D is not likely an antagonistic competitive strategy.

**FIGURE 5:**
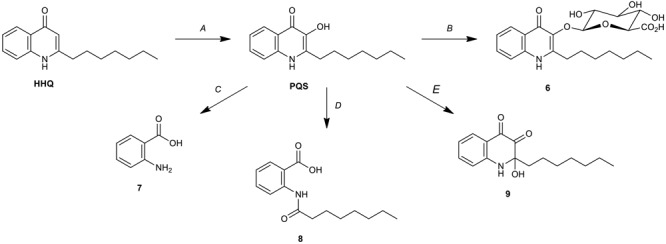
Microbial biotransformations of HHQ/PQS. **(A)** Oxidation of HHQ by *P. aeruginosa* (PqsH) (Gallagher et al., 2002), *Rhodococcus* sp. BG43 and *Streptomyces* sp. RKBH-B178. **(B)** Glucuronidation of PQS by *Streptomyces* sp. RKBH-B178. **(C)** Degradation of PQS to anthranilic acid **(7)** by *Rhodococcus* sp. BG43 (Muller et al., 2014). **(D)** Degradation of PQS to N-octanoylanthranilic acid **(8)** by *Arthrobacter nitroguajacolicus* strain Rü61a (Hod) (Pustelny et al., 2009). **(E)** Oxidation of PQS to HHQD **(9)** by *Achromobacter xylosoxidans* Q19 (Soh et al., 2015).

The discovery of PQS-GlcA (**6**) represents an unprecedented biotransformation of HHQ/PQS by *Streptomyces* sp. RKBH-B178. Thus, RKBH-B178 is likely to perform a two-step biotransformation to produce PQS-GlcA. Using HHQ as the substrate, the biotransformation requires an oxidation at C-3 of the quinolone ring, followed by glucuronidation of the resulting hydroxyl (Figures 5A,B). There are only three known microbial biotransformation mechanisms which deactivate HHQ/PQS. *Rhodococcus* sp. BG43 degrades PQS and HHQ to anthranilate (**7**) (Figure 5C; Muller et al., 2014). In this biotransformation, HHQ is first oxidized to PQS before further modification, which is consistent with the first step of our proposed biotransformation of PQS-GlcA. In a second example, 1-H-3-hydroxy-4-oxoquinaldine-2,4-dioxygenase (Hod), whose natural substrate is 3-hydroxy-2-methyl-4(1*H*)-quinolone, was shown to oxidize PQS, which is structurally similar to the natural substrate of Hod (Pustelny et al., 2009). Addition of Hod to cultures of *P. aeruginosa* reduced the expression of *pqsA* and related virulent factors, suggesting the oxidized product (**8**) had only weak quorum sensing activity (Figure 5D; Pustelny et al., 2009). Thirdly, PQS is oxidized to 2-heptyl-2-hydroxy-1,2-dihydroquinoline-3,4-dione (HHQD, **9**) by *Achromobacter xylosoxidans* (Figure 5E; Soh et al., 2015). To the best of our knowledge, biotransformation of HHQ/PQS to PQS-GlcA is just the fourth example of a biotransformation of HHQ/PQS, and the first reported glycosylation of PQS.

Glucuronidation is one of the mechanisms bacteria use to deactivate antibiotics (Wright, 2005), and has been reported in *S. lividans* (Cundliffe, 1992) and *Nocardia species* (Morisaki et al., 1993). Of the 14 actinomycetes tested (Supplementary Table S4) for the ability to perform this modification, only RKBH-B178 possessed this trait, suggesting glucuronidation of HHQ/PQS is not common among actinomycetes, although, a larger number of strains would need to be tested to more fully evaluate the distribution. Under the cultivation conditions utilized in this study RKBH-B178 generated 0.4 mg (0.9 μmol) of PQS-GlcA from 2.95 L of culture supplemented with 17.9 mg (73.6 μmol) of HHQ, representing a 1.2% conversion level. This low conversion level may be due to suboptimal culture conditions, resulting in low expression levels of the GlcA transferase or low concentrations of reaction substrates. Alternatively, the low conversion level may indicate that these quinolones are not the natural substrates of the glucuronidating enzyme. Analysis of the RKBH-B178 genome and characterization of glycosyltransferases encoded therein will be required to biochemically characterize the HHQ-glucuronidating enzyme possessed by this strain.

*Pseudomonas aeruginosa* is a ubiquitous environmental bacterium and opportunistic pathogen (Winstanley et al., 2016). The group behavior of *P. aeruginosa* is coordinated via a complex quorum sensing network consisting of four components: the *las* (Gambello and Iglewski, 1991; Jones et al., 1993; Pearson et al., 1994), *rhl* (Ochsner and Reiser, 1995; Pearson et al., 1995), *pqs* (Pesci et al., 1999; Cao et al., 2001; Gallagher et al., 2002), and *iqs* (Lee et al., 2013) systems. The *pqs* system is an important component of *Pseudomonas aeruginosa* quorum sensing, contributing to the regulation of biofilm formation and production of virulence factors such as pyocyanin, elastase, rhamnolipids and PA-IL lectin (Diggle et al., 2006). The receptor for PQS is PqsR, a LysR-type transcription regulator that also controls expression of the PQS biosynthetic operon (*pqsABCDE*) by directly binding to its promoter (Cao et al., 2001). We have demonstrated that *Streptomyces* sp. RKBH-B178 is able to biotransform HHQ and PQS to a glycosylated derivative that is unable to bind to PqsR and drive gene expression from the PQS operon promoter. It is tempting to speculate that *Streptomyces* sp. RKBH-B178 uses this biotransformation mechanism to compete with *P. aeruginosa* or other species utilizing a *pqs*-based quorum sensing system. Further studies of the interactions between *Streptomyces* RKBH-B178 and gram-negative bacteria utilizing HHQ/PQS quorum sensing and identification of the glucuronidating enzyme would be needed to elucidate the competitive advantage afforded by biotransformation of HHQ/PQS.

In conclusion, the bacterial culture strategy presented herein is a promising tool to identify chemical signaling between microbes and for natural product discovery. Our screening using seven producers and four inducers demonstrated induction of a range of putatively novel natural products. The strategy enables preparation of large quantities of autoclaved inducer culture stocks which can be used in both small-scale screening and for large-scale production of the target natural product. As two examples of the efficacy of the described bacterial culture approach, this report describes the discovery of a new natural product, hydrazidomycin D, and the identification of the new HHQ/PQS biotransformation product PQS-GlcA representing a new deactivation mechanism of this quorum sensing system.

## Author Contributions

LL, AS, BH, FB, DM, DO, and RK designed the experiments. LL performed the metabolomics studies. HC operated the UHPLC-HRESIMS instrumentation. LL, AS, and DM performed the purification and structure elucidation. LL and BH performed the molecular docking and beta-glucosidase assays. ND performed the screening of HHQ for general effects in 14 *Streptomyces*. KM performed the cytotoxicity assays. LL, and ML performed antimicrobial assays. LL, BH, DM, and RK contributed to the final version of the manuscript.

## Conflict of Interest Statement

The authors declare that the research was conducted in the absence of any commercial or financial relationships that could be construed as a potential conflict of interest.
