# Supplementary material for: Discovery of a New Natural Product and a Deactivation of a Quorum Sensing System by Culturing a “Producer” Bacterium With a Heat-Killed “Inducer” Culture
Source: Front Microbiol. 2019 Jan 17;9:3351. doi: 10.3389/fmicb.2018.03351 (PMC6344404; doi:10.3389/fmicb.2018.03351)
Supplement: Supplementary file 1 [file Data_Sheet_1.pdf]

Supplementary Material

Discovery of a New Natural Product and a Deactivation of a Quorum Sensing System by Culturing a "Producer" Bacterium with a Heat-Killed "Inducer" Culture

Libang Liang[1], Amanda Sproule[1], Brad Haltli[1,2,3], Douglas H. Marchbank[1,3], Fabrice Berrué[1], David P. Overy[1,4], Kate McQuillan[3], Martin Lanteigne[1], Noelle Duncan[3], Hebelin Correa[3] and Russell G. Kerr[1,2,3]*

[1] Department of Chemistry, University of Prince Edward Island, Charlottetown, PE, Canada,
[2] Department of Biomedical Sciences, Atlantic Veterinary College, University of Prince Edward Island, Charlottetown, PE, Canada,
[3] Nautilus Biosciences Croda, Charlottetown, PE, Canada,
[4] Department of Pathology and Microbiology, Atlantic Veterinary College, University of Prince Edward Island, Charlottetown, PE, Canada

***Correspondence:** Russell G. Kerr: rkerr@upei.ca

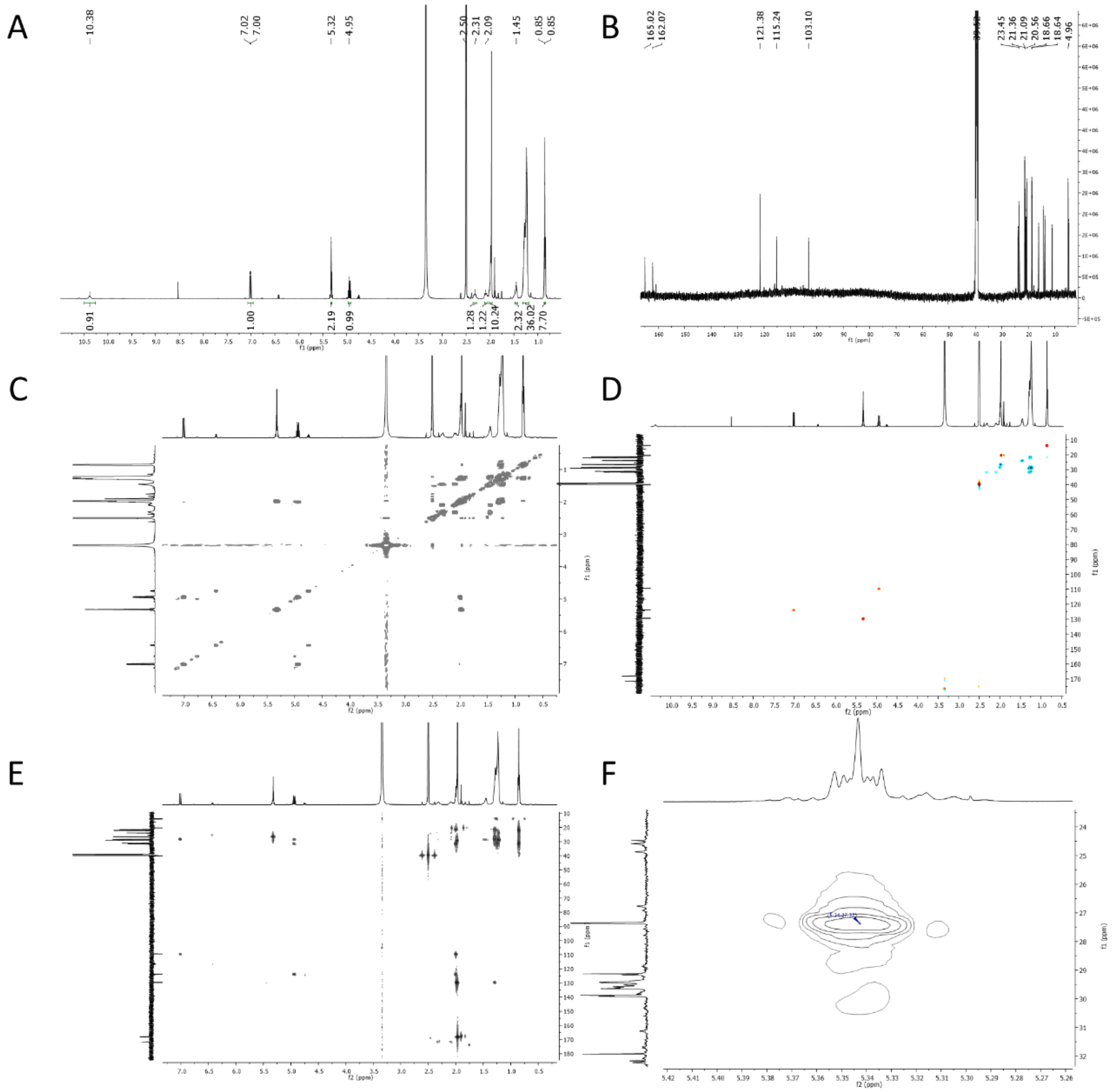

Supplementary Figure S1 | 1D and 2D (^{1}H 600 MHz, ^{13}C 150 MHz) NMR spectra of hydrazidomycin D in DMSO-*d_6* and $CDCl_3$.

(A)-(E) are ^{1}H, ^{13}C, COSY, ^{1}H-^{13}C HSQC (red cross-peaks are negatively phased, blue cross-peaks are positively phased), ^{1}H-^{13}C HMBC (coupling constant 5 Hz) NMR spectra in DMSO-d_6, respectively. **(F)** is ^{1}H-^{13}C HMBC NMR spectrum (coupling constant 5 Hz) in $CDCl_3$. The α-carbons to the double bond in the C18 alkyl chain had chemical shifts of 27.37 ppm, suggesting a *cis*-configuration of the double bond. $CDCl_3$ was chosen as a solvent for direct chemical shift comparison with data reported by (van Boom et al., 1977).

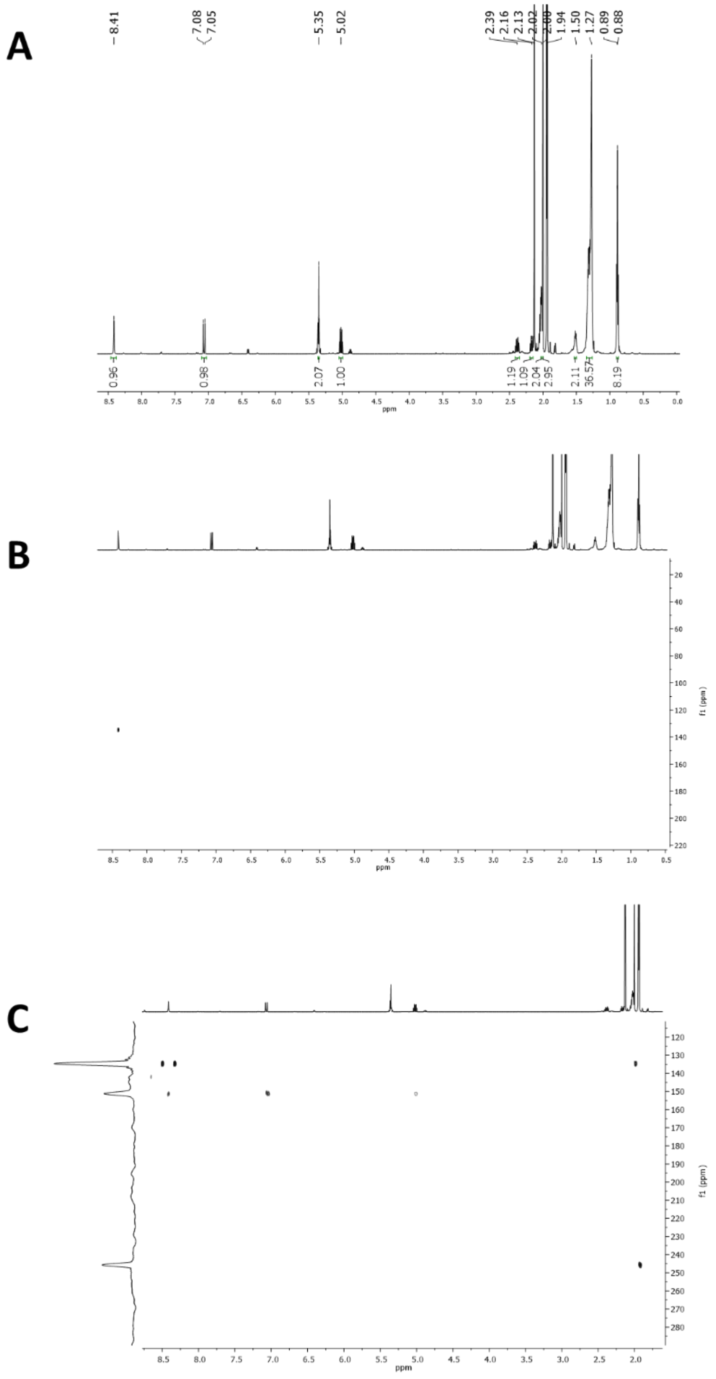

Supplementary Figure S2 | 1D and 2D (^{1}H 600 MHz, ^{15}N 60 MHz) NMR spectra of hydrazidomycin D in CD_3CN.

(A)-(C) are ^{1}H, ^{1}H-^{15}N HSQC (coupling constant 90 Hz), ^{1}H-^{15}N HMBC (coupling constant 8 Hz) NMR spectra in CD_3CN, respectively.

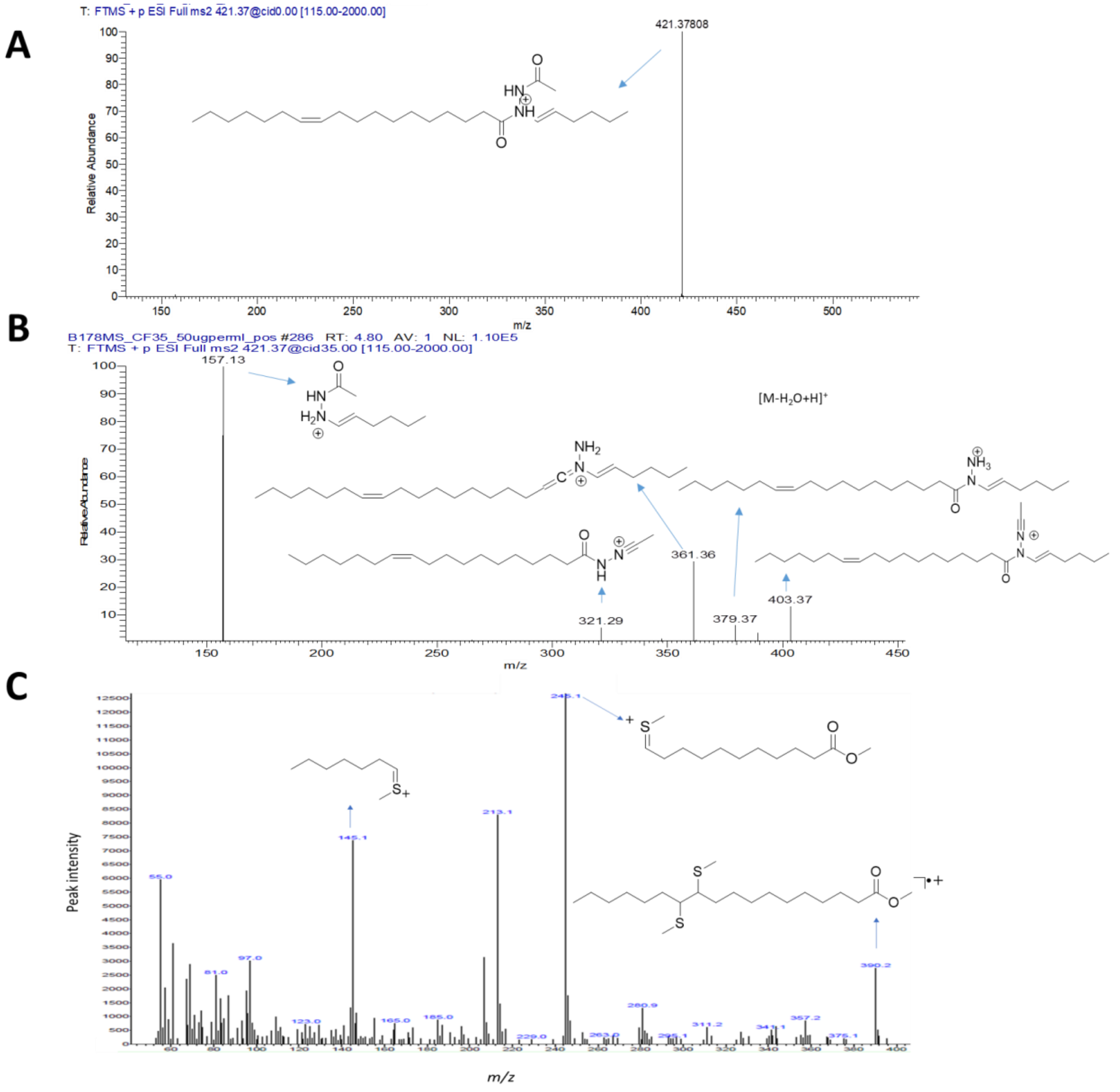

Supplementary Figure S3 | Mass spectrometry characterization of hydrazidomycin D.
(A) High-resolution mass spectrum (MS^1) in ESI positive mode of hydrazidomycin D showing the parent *m/z* 421.3781 $[M + H]^+$ adduct. **(B)** MS/MS spectrum (35 eV) in ESI positive mode of hydrazidomycin D with proposed fragment structures. **(C)** GC-EIMS spectrum of the DMDS derivatized product confirming the double bond is located between C11 and C12.

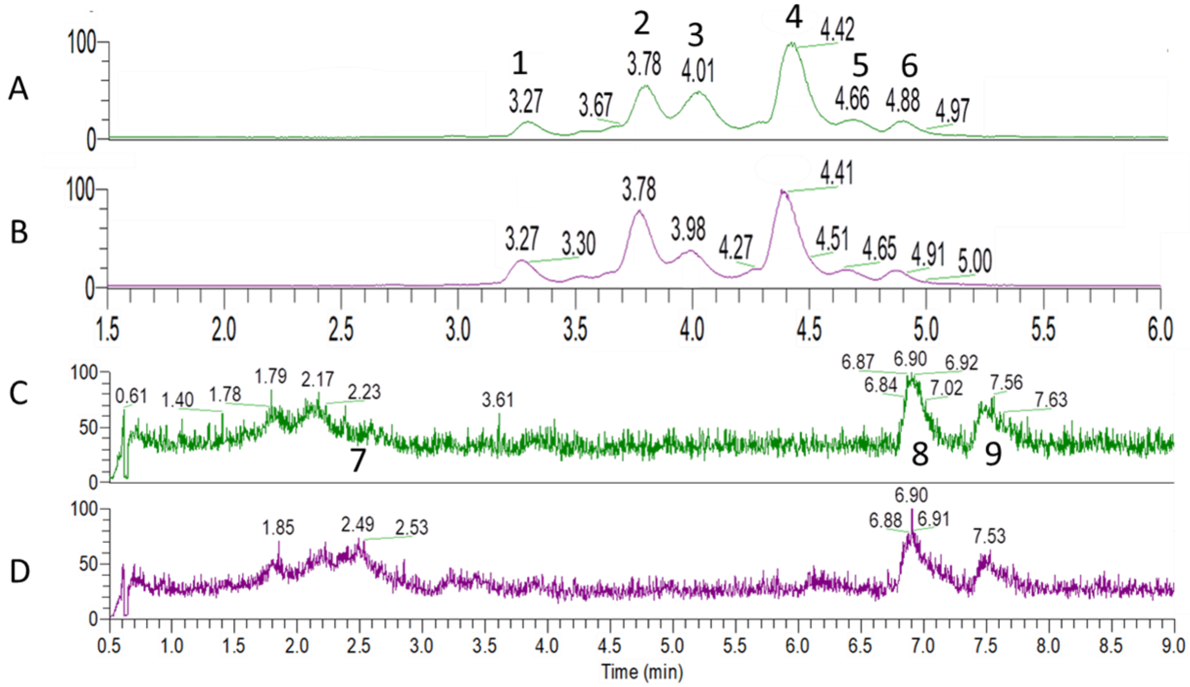

Supplementary Figure S4 | Comparison of UHPLC-HRESIMS profiles *P. aeruginosa* (ISP2, 30°C, 200 rpm, 48 h) and *M. smegmatis* liquid cultures (ISP2, 30°C, 200 rpm, 144 h) before and after autoclaving.

Chromatograms **(A)**, **(B)**, **(C)**, and **(D)** are autoclaved *P. aeruginosa*, non-autoclaved *P. aeruginosa*, autoclaved *M. smegmatis*, and non-autoclaved *M. smegmatis* cultures, respectively. The maximum peak intensity of chromatograms **(A)** – **(D)** are 7.5E6, 5.8E6, 3.7E5, and 4.6E5, respectively.

In **(A)** and **(B)**, peak 1 is HHQ (*m/z* 244.1696 $[M + H]^+$, calcd 244.1696); peak 2 is a putative HHQ analogue with a mass difference of two methylene groups (*m/z* 272.2006 $[M + H]^+$, calcd 272.2009; peak 3 is Rha-Rha-C10-C10 (*m/z* 673.3778 $[M + Na]^+$, calcd 673.3770); and peak 4 is Rha-C10-C10 (*m/z* 527.3192 $[M + Na]^+$, calcd 527.3191); peaks 5 and 6 are putative Rha-C10-C10 analogues (*m/z* 553.3360 $[M + Na]^+$, calcd 553.3347 and *m/z* 555.3515 $[M + Na]^+$, calcd 555.3504).

In **(C)** and **(D)**, peaks between 1.5 min to 3 min are mainly media components from ISP2, except peak 7 is a *M. smegmatis* metabolite (*m/z* 247.10780 $[M + H]^+$); peak 8 is the added 10 μg/mL internal standard dioctyl phthalate (*m/z* 391.28427 $[M + H]^+$, calcd 391.28429); peak 9 is a *M. smegmatis* metabolite (*m/z* 338.34161 $[M + H]^+$).

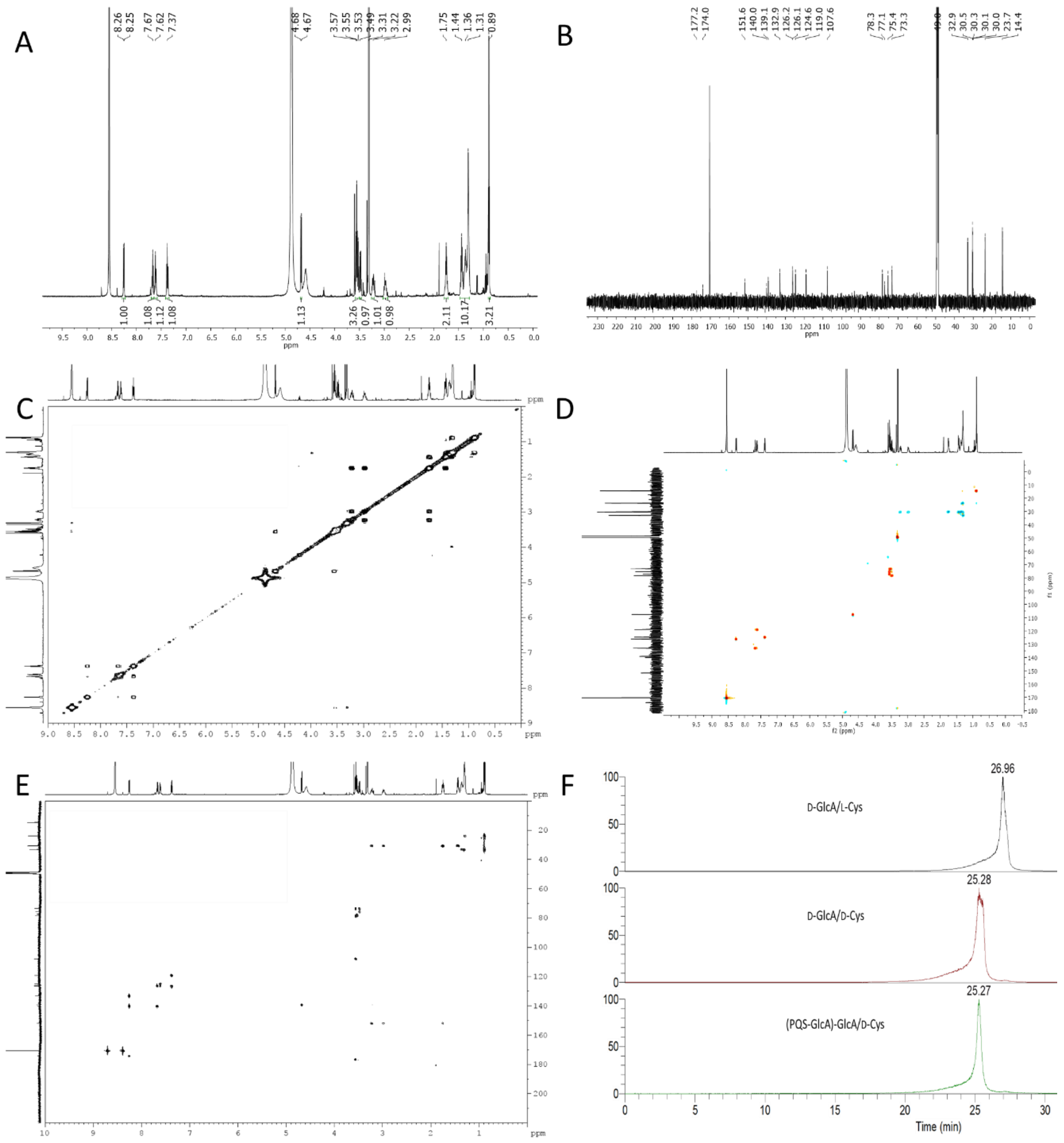

Supplementary Figure S5 | 1D and 2D (^{1}H 600 MHz, ^{13}C 150 MHz) NMR spectra of PQS-GlcA in CD_3OD and chromatographic characterization of stereochemistry of the glucuronic acid moiety.

(A)-(E) are ^{1}H, ^{13}C, COSY, ^{1}H-^{13}C HSQC (red cross-peaks are negatively phased, blue cross-peaks are positively phased), ^{1}H-^{13}C HMBC (coupling constant 5 Hz) NMR spectra in CD_3OD, respectively. **(F)** is UHPLC-HRESIMS chromatograms on D-GlcA/L-cysteine methyl ester, D-GlcA/D-cysteine methyl ester and PQS-GlcA/D-cysteine methyl ester, respectively. Retention time measurement L-GlcA/D-Cys was by its enantiomer D-GlcA/L-Cys. This result suggests the glucuronic acid moiety in PQS-GlcA have a D-configuration.

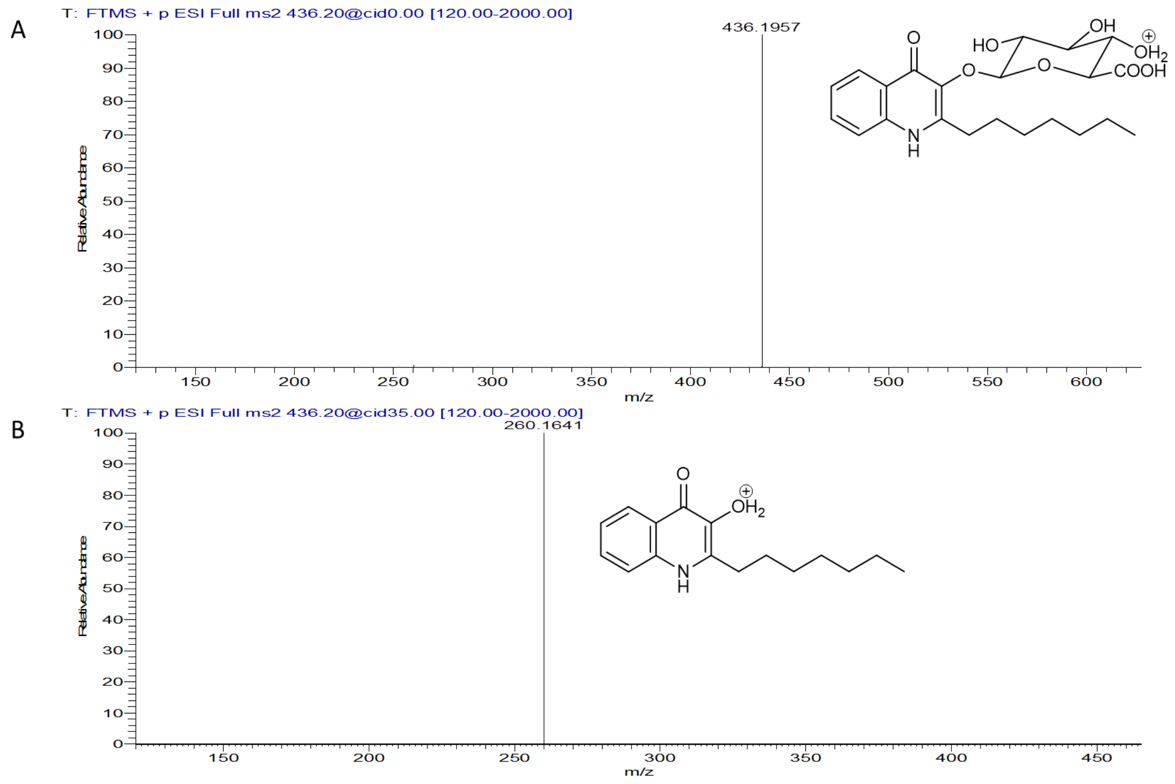

Supplementary Figure S6 | ESI tandem mass spectrometry in positive mode of PQS-GlcA.

(A) High-resolution mass spectrum (MS^1) of PQS-GlcA showing the parent *m/z* 436.1957 $[M + H]^+$ adduct. **(B)** MS/MS spectrum (35 eV) of PQS-GlcA with fragment ion *m/z* 260.1641 $[M + H]^+$ that is consistent with the PQS aglycone portion of PQS-GlcA.

Supplementary Table S1 | Possible identities of the mass features of interest based on a search of AntiBase 2017. These compounds were selected based on induction, up-regulation and/or production level in culture screening.

Mass feature (m/z_RT)	Adduct	Metabolomics	Producer	Inducer	Database match (<5 ppm)	Δppm	Source Organism	Reference
368.1493_4.19		U	VI	AF_A				
382.165_4.41		U	VI	AF_A				
258.1125_2.66	$[M+H]^+$	U	VI	AF_A	carbazomycin G	0.071	*Streptoverticillium ehimense*	(Kaneda et al., 1988)
416.1493_4.35	$[M+Na]^+$	U	VI	AF_A	latrunculin T	2.198	Sponge	(El Sayed et al., 2006)
421.3781_5.79		U	VI	MS_A, MS_B, PA_B				
313.2848_4.62		I	VI	MS_A, MS_B, PA_B				
271.0602_3.59	$[M+H]^+$	U	VI	PA_B	3,8-dihydroxy-1-methoxy-9,10-anthraquinone	0.369	*Xenorhabdus luminescens*	(Richardson et al., 1988)
221.1262_2.46	$[M+Na]^+$	U	VI	PA_B	Maniwamycin A	1.80	*Streptomyces prasinopilosus*	(Takahashi et al., 1989)
467.2079_2.81	$[M+H]^+$	U	VI	BS_A	Staurosporine	0.284	*Streptomyces griseolus*	(Funato et al., 1994)
455.1604_4.15		I	VI	AF_A				
441.1447_4.06		I	VI	AF_A				
240.102_2.66	$[M+H]^+$	I	VI	AF_A	Aspergilitine	0.395	*Aspergilus versicolor*	(Lin et al., 2003)
314.1023_1.64		I	VI	AF_A				
240.1022_3.08	$[M+H]^+$	I	VI	AF_A, MS_A	Aspergilitine	0.395	*Aspergilus versicolor*	(Lin et al., 2003)
432.1443_3.77		U	VI	AF_A				
588.3439_1.93		U	VI	AF_A, PA_B				
436.1966_2.75		I	VI	PA_B				
435.3947_6.00		I	VI	MS_A				
789.7225_5.72		I	VI	MS_A				
254.0811_2.05	$[M+H]^+$	I	VI	BS_A, MS_A	utahmycin A	0.274	*Streptomyces albus* J1704 + metagenoic DNA	(Bauer et al., 2010)
263.1767_3.22		U	VI	BS_B				
404.3161_5.30		I	III	AF_A, BS_B, MS_A				
224.0919_1.71	$[M+H]^+$	I	III	AF_A	Christolane C	0.739	*Streptomyces lydicus* NRRL 2433 mutants	(Gómez et al., 2012)
275.0551_2.70	$[M+H]^+$	U	VII	MS_A, MS_B	Juglomycin A/B	0.311	*Streptomyces diastatochromogenes*	(牛山敬一 et al., 1971)
389.2324_4.75		I	I	BS_B, MS_A, MS_B				
1087.4727_4.21	$[M+H]^+$	U	V	BS_B, MS_A, MS_B	Landomycin A	1.838	*Streptomyces* sp.	(Henkel et al., 1990)
1109.4521_4.20		U	V	BS_B, MS_A, MS_B				
1104.4989_4.21		U	V	BS_B, MS_A, MS_B				

U: up-regulated; I: induced.

Supplementary Table S2 | ^{1}H (600 MHz) and ^{13}C (150 MHz) NMR data for hydrazidomycin D recorded in DMSO-d_6.

Position	δ_C/δ_N , type	δ_H, (J in Hz)	COSY	HMBC (H → C)
1	171.6, C	-	-	-
2	31.6, CH_2	2.31, 2.09, m	3	C-1, 3
3	23.9, CH_2	1.45, m	2, 4	C-1[a], 2[a]
4	28.3~29.0[b], CH_2	1.20, m	3	-
5	28.3~29.0[b], CH_2	1.21~1.29 [b], m	-	-
6	28.3~29.0[b], CH_2	1.21~1.29 [b], m	-	-
7	28.3~29.0[b], CH_2	1.21~1.29 [b], m	-	-
8	28.3~29.0[b], CH_2	1.26, m	9	-
9	29.1, CH_2	1.29, m	8, 10	-
10	26.6, CH_2	1.97, m	9, 11	9, 12
11	129.7, CH	5.32, dt (9.6, 5.1)	10, 12	9, 13
12	129.7, CH	5.32, dt (9.6, 5.1)	11, 13	10, 14
13	26.6, CH_2	1.97, m	12, 14	11, 14
14	29.1, CH_2	1.29, m	13, 15	-
15	28.3~29.0[b], CH_2	1.26, m	14	-
16	31.1, CH_2	1.24, m	-	-
17	21.5, CH_2	1.26, m	18	-
18	13.8, CH_3	0.85, t (7.0)	17	C-16, 17
19	123.9, CH	7.01, d (14.1)	20	C-20, 21, N-A, N-B
20	109.6, CH	4.94, dt (14.1, 7.1)	19,21	C-19, 21, 22, N-B
21	28.6, CH_2	1.99, m	20, 22	C-19, 20, 22, 23
22	31.6, CH_2	1.28, m	22	-
23	21.5, CH_2	1.26, m	24	-
24	13.9, CH_3	0.85, t (7.1)	23	C-22, 23
25	168.1, C	-	-	-
26	20.4, CH_3	1.97, s	-	C-25, N-A
N^{α}	134.6[c], NH	10.38, s (broad), 8.41[c], s	-	N-B[c], C-25
N^{β}	151.2[c], N	-	-	-

a. correlations observed in CD_3OD. b. signals are not distinguishable. c. chemical shifts and correlations measured in CD_3CN.

Supplementary Table S3 | ^{1}H (600 MHz) and ^{13}C (150 MHz) NMR data for PQS-GlcA recorded in CD_3OD.

Position	δ_C, type	δ_H, (*J* in Hz)	COSY	HMBC (H→C)
1	-	12.32*, s (broad)	-	-
2	151.6, C	-	-	-
3	139.1, C	-	-	-
4	174.0, C	-	-	-
5	126.1, C	-	-	-
6	126.2, CH	8.27, d (8.1)	7	4, 8, 10
7	124.6, CH	7.37, dd (7.5, 8.1)	6, 7	5, 9
8	132.9, CH	7.67, dd (7.5, 8.5)	7, 9	6, 10
9	119.0, CH	7.62, d (8.5)	8	5, 7
10	140.0, C	-	-	-
11	30.5, CH_2	3.22, m; 2.99, m	11, 12	2, 3, 12
12	30.0, CH_2	1.75, quint (7.7)	11, 13	11, 13
13	30.1, CH_2	1.44, m; 1.36, m	12, 13, 14	12, 14
14	30.3, CH_2	1.31, m	13	13, 15
15	32.9, CH_2	1.30, m	-	14, 16
16	23.7, CH_2	1.32, m	17	15, 17
17	14.4, CH_3	0.89, t (6.8)	16	15, 16
1'	107.6, CH	4.68, d (7.8)	2'	3
2'	75.4, CH	3.55, dd (7.8, 8.7)	1', 3'	-
3'	78.3, CH	3.49, dd (8.7, 8.7)	2', 4'	-
4'	73.3, CH	3.53, dd (8.7, 9.5)	3', 5'	-
5'	77.1, CH	3.57, d (9.5)	4'	6'
6'	177.2, C	-	-	-

*Measured in DMSO-d_6

Supplementary Table S4 | Identities of 14 environmental actinomycete strains tested for secondary metabolite induction in response to HHQ exposure.

GenBank accession numbers are provided for 16S rRNA gene sequences of the strains used in this study. The % ID value is that of the highest scoring match in a BlastN search of the 16S rRNA sequence database.

Strain	GenBank Accession No.	Closest BlastN Hit (Accession No.)	% ID
RKBH-B092	KY362380	*Streptomyces tateyamensis* (NR_112842)	99.80
RKBH-B124	KY362382	*Streptomyces rochei* (NR_116078)	100.00
RKBH-B476	KY362385	*Streptomyces gelaticus* (NR_043488)	98.05
RKBH-B477	KY362386	*Streptomyces galilaeus* (NR_040857)	98.86
RKHS-054	KY362387	*Streptomyces bellus* (NR_041222)	97.51
RKHS-126	KY362388	*Streptomyces bikiniensis* (NR_026177)	99.27
RKHS-142	KY362389	*Promicromonospora iranensis* (NR_109446.1)	98.92
RKHS-146	KY364190	*Streptomyces ederensis* (NR_112457)	99.06
RKLL-001	KY362390	*Streptomyces fulvissimus* (NR_103947)	99.87
RKND-081	KY362391	*Streptomyces badius* (NR_043350)	99.53
RKND-187	KY362392	*Streptomyces drozdowiczii* (NR_116093)	99.46
RKND-216	KY362393	*Streptomyces qinglanensis* (NR_109303)	98.92
RKND-444	KY362394	*Streptomyces zaomyceticus* (NR_044144)	99.93
RKNM-081	KY362396	*Streptomyces canus* (NR_043347)	98.93

Supplementary Table S5 | Biological activities of some nitrogen-nitrogen bond containing natural products.

natural products	biological activities	source	references
Hydrazidomycin A	IC_{50} of 0.857μM on average against 42 human tumor cells (0.37μM on average of 12 cell lines)	*Streptomyces atratus*	(Ueberschaar et al., 2011)
Hydrazidomycin B	IC_{50} of 10.7μM on average against 42 human tumor cells (6.04 μM on average of 12 cell lines)	*Streptomyces atratus*	(Ueberschaar et al., 2011)
Geralcin B	IC_{50} of 5μM against MDA231 breast cancer cells	*Streptomyces* sp. LMA-545	(Le Goff et al., 2012)
Geralcin C	IC_{50} of 0.8μM against KB and HCT116 cancer cells, 0.7mM against E. coli DnaG primase	*Streptomyces* sp. LMA-545	(Le Goff et al., 2013)
Elainomycin	0.24-1.25μg/ml against *M. tuberculosis*, IC_{50} of 16.3μM against HepG2 cancer cells	*Streptomyces hepaticus*	(Ehrlich et al., 1954, Karlson, 1962)
	IC_{50} of 12.26μM on average against 12 human tumor cells	*Streptomyces sp. strain HKI0708*	*(Ding et al., 2012)*
Elainomycin B	IC_{50} of 100μM, 1.0µM, 6.5µM against *S. lentus*, acetycholinesterase, phosphodiesterase	*Streptomyces* sp. BK 190	(Helaly et al., 2011)
Elainomycin C	IC_{50} of 100μM, 2.0µM, 8µM against *S. lentus*, acetycholinesterase, phosphodiesterase	*Streptomyces* sp. BK 190	(Helaly et al., 2011)
Elainomycin H	IC_{50} of 4.86μM on average against 12 human tumor cells	*Streptomyces* sp. strain HKI0708	*(Ding et al., 2012)*
Elainomycin K	IC_{50} of 30.05μM, 54.15µM, 47.5µM against *B. subtilus*, S. *lentus*, X. *camperstris*	*Streptomyces* sp. Tü 6399	(Manderscheid et al., 2013)
Elainomycin L	IC_{50} of 22.9μM, 41.7µM, 51.3µM against *B. subtilus*, *S. lentus*, *X. camperstris*	*Streptomyces* sp. Tü 6399	(Manderscheid et al., 2013)
LL-BH872α	potent antifungal agent	*Streptomyces hinnulinus*	(McGahren and Kunstmann, 1969)
Valanimycin	MIC 0.078μg/ml, 1.25μg/ml, 1.25μg/ml against *E. coli* BE1121, *E. coli* K12, *E. coli* ML1629, IC_{50} of 0.79μg/ml, 1.44μg/ml, 2.65μg/ml against L1210, P388/ADR, P388/S leukemia cells, and prolonged life span of mice inoculated with Ehrlich carcinoma or L1210	*Streptomyces viridifaciens* MG456-hF10	(Amato et al., 1986)
Jietacins A	0.25μg/ml exhibited 100% mortality *Bursaphelenchus lignicolus*, 11 mm and 7.5 mm zone of inhibition against *A. niger* and *M. racemosus* at concentration of 1.0mg/ml	*Streptomyces* sp. KP-197	(Omura et al., 1987)
Jietacins B	0.25μg/ml exhibited 100% mortality *Bursaphelenchus lignicolus*	*Streptomyces* sp. KP-197	(Omura et al., 1987)
Lyophyllin	tumor inhibiting	*Lyophyllum connatum*	(Fugmann and Steglich, 1984)
Maniwamycins A	8 mm zone of inhibition (50μg/disk) against Rhodotorula sp.	*Actinomadura* sp.	(Takahashi et al., 1989)

REFERENCES

Amato M., Iinuma H., Naganawa H., Yamagishi Y., Amada M., Masuda T., et al. (1986). Isolation and properties of valanimycin, a new azoxy antibiotic. *J. Antibiot.* 39, 184-91.

Bauer J. D., King R. W., Brady S. F. (2010). Utahmycins A and B, azaquinones produced by an environmental DNA clone. *J. Nat. Prod.* 73, 976-9. doi: 10.1021/np900786s

Ding L., Ndejouong, Basile Le Sage Tchize, Maier A., Fiebig H., Hertweck C. (2012). Elaiomycins D–F, antimicrobial and cytotoxic azoxides from Streptomyces sp. strain HKI0708. *J. Nat. Prod.* 75, 1729-34. doi: 10.1021/np300329m

Ehrlich J., ANDERSON L. E., Coffey G., Feldman W., Fisher M., Hillegas A., et al. (1954). Elaiomycin, a New Tuberculostatic Antibiotic. Biologic Studies. *Antibiotics & Chemotherapy* 4, 338-42.

El Sayed K. A., Youssef D. T., Marchetti D. (2006). Bioactive natural and semisynthetic latrunculins. *J. Nat. Prod.* 69, 219-23. doi: 10.1021/np050372r

Fugmann B., and Steglich W. (1984). Unusual components of the toadstool Lyophyllum connatum (agaricales). *Angewandte Chemie International Edition* 23, 72-3.

Funato N., Takayanagi H., Konda Y., Toda Y., Harigaya Y., Iwai Y., et al. (1994). Absolute configuration of staurosporine by X-ray analysis. *Tetrahedron Lett.* 35, 1251-4.

Gómez C., Olano C., Palomino-Schätzlein M., Pineda-Lucena A., Carbajo R. J., Brana A. F., et al. (2012). Novel compounds produced by Streptomyces lydicus NRRL 2433 engineered mutants altered in the biosynthesis of streptolydigin. *J. Antibiot.* 65, 341-8. doi: 10.1038/ja.2012.37

Helaly S. E., Pesic A., Fiedler H., Süssmuth R. D. (2011). Elaiomycins B and C: Alkylhydrazide antibiotics from Streptomyces sp. BK 190. *Org. Lett.* 13, 1052-5. doi: 10.1021/ol1031014

Henkel T., Rohr J., Beale J. M., Sshwenen L. (1990). Landomycins, new angucycline antibiotics from Streptomyces sp. *J. Antibiot.* 43, 492-503.

Kaneda M., Naid T., Kitahara T., Nakamura S., Hirata T., Suga T. (1988). Carbazomycins G and H, novel carbazomycin-congeners containing a quinol moiety. *J. Antibiot.* 41, 602-8.

Karlson A. (1962). Specific inhibitory effect of elaiomycin in vitro upon Mycobacterium tuberculosis. *Antibiotics & Chemotherapy* 12, 446-9.

Le Goff G., Martin M., Iorga B. I., Adelin E., Servy C., Cortial S., et al. (2013). Isolation and Characterization of Unusual Hydrazides from Streptomyces sp. Impact of the Cultivation Support and Extraction Procedure. *J. Nat. Prod.* 76, 142-9. doi: 10.1021/np300527p

Le Goff G., Martin M., Servy C., Cortial S., Lopes P., Bialecki A., et al. (2012). Isolation and Characterization of α, β-Unsaturated γ-Lactono-Hydrazides from Streptomyces sp. *J. Nat. Prod.* 75, 915-9. doi: 10.1021/np300026p

Lin W., Brauers G., Ebel R., Wray V., Berg A., Sudarsono, et al. (2003). Novel Chromone Derivatives from the Fungus Aspergillus versicolor Isolated from the Marine Sponge Xestospongia exigua. *J. Nat. Prod.* 66, 57-61. doi: 10.1021/np020196b

Manderscheid N., Helaly S. E., Kulik A., Wiese J., Imhoff J. F., Fiedler H., et al. (2013). Elaiomycins K and L, new azoxy antibiotics from Streptomyces sp. Tü 6399. *J. Antibiot.* 66, 85-8. doi: 10.1038/ja.2012.99

McGahren W. J., and Kunstmann M. (1969). Novel. alpha.,. beta.-unsaturated azoxy-containing antibiotic. *J. Am. Chem. Soc*. 91, 2808-10.

Omura S., Otoguro K., Imamura N., Kuga H., Takahashi Y., Masuma R., et al. (1987). Jietacins A and B, new nematocidal antibiotics from a Streptomyces sp. *J. Antibiot.* 40, 623-9.

Richardson W. H., Schmidt T. M., Nealson K. H. (1988). Identification of an anthraquinone pigment and a hydroxystilbene antibiotic from Xenorhabdus luminescens. *Appl. Environ. Microbiol.* 54, 1602-5.

Takahashi Y., Nakayama M., Watanabe I., Deushi T., Ishiata H., Shiratsuchi M., et al. (1989). Novel Antifungal Antibiotics Maniwamycins A and B. *J. Antibiot.* 42, 1541-6.

Ueberschaar N., Ndejouong, Ble S., Ding L., Maier A., Fiebig H., Hertweck C. (2011). Hydrazidomycins, cytotoxic alkylhydrazides from Streptomycesatratus. *Bioorg. Med. Chem. Lett.* 21, 5839-41. doi: 10.1016/j.bmcl.2011.07.108

Van Boom J., Burgers P., Haasnoot C., Reese C. (1977). Recueil des Travaux Chimiques des Pays-Bas Journal of the Royal Netherlands Chemical Society: General method for the synthesis of 3′, 5′-diesters and 2′-acetals of the four common nucleosides. *Recueil des Travaux Chimiques des Pays-Bas* 96, 91-5.

牛山敬一, 田中信寿, 小野博史, 尾形晴見. (1971). 新抗生物質 Juglomycins の研究 第 1 報. *Jpn J. Antibiot.* 24, 197-9.
